# A novel Weighted Exponential-Lindley distribution with Bayesian estimation under progressive Type-II censoring and a Bayesian-Calibrated Variational Autoencoder for survival prediction

**DOI:** 10.3389/frai.2026.1934204

**Published:** 2026-10-07

**Authors:** P. Furqan Ahmed, V. Sujatha

**Affiliations:** 1 Department of Mathematics, School of Advanced Sciences, Vellore Institute of Technology, Vellore, Tamil Nadu, India; 2 Department of Quantum AI, School of Computer Science and Engineering, Vellore Institute of Technology, Vellore, Tamil Nadu, India

**Keywords:** Bayesian estimation, Exponential-Lindley mixture, hazard rate, MCMC, progressive Type-II censoring, reliability engineering, survival analysis, variational autoencoder

## Abstract

One-parameter lifetime models, such as the exponential and Lindley distributions, and two-parameter extensions such as the weighted Lindley distribution cannot separately control tail weight and dispersion, since a single shape parameter governs both. We address this with the Weighted Exponential-Lindley (WEL) distribution, a three-parameter model built by applying a length-biased weight to a mixture of exponential and Lindley components, with closed-form density, survival function, and an exact two-component gamma mixture representation. Using this representation and Glaser's failure-rate criterion, we prove that the WEL hazard rate is strictly increasing on its entire parameter space, with the scale parameter as its asymptotic level; the weight parameter then gives independent, tunable control over dispersion, skewness, and kurtosis are unavailable in the Lindley or weighted Lindley families. We develop maximum likelihood, method-of-moments, and Bayesian estimation under progressive Type-II censoring, validated through a Monte Carlo study across several parameter configurations and sample sizes, reporting bias, MSE, interval coverage, computation time, and convergence rates, including non-convergent method-of-moments runs rather than only successful ones. We then propose the Bayesian-Calibrated Variational Autoencoder Survival Model (BC-VAESM), whose decoder outputs individual, covariate-dependent WEL parameters, trained end-to-end via a censored negative log-likelihood. We evaluate both contributions on real, publicly available survival datasets, including censored clinical trial data and complete reliability/biological benchmarks, one chosen specifically to illustrate WEL's inability to represent bathtub hazards and one matching its increasing-hazard property. Fitting the censored datasets under an explicit right-censored likelihood, distinguished from the progressive Type-II scheme used in the Bayesian machinery, WEL is compared against an expanded set of competing one-, two-, and three-parameter lifetime models, including further weighted distributions. WEL is the best-fitting model on one dataset but is outperformed on others, and we report all outcomes without selective emphasis. In five-fold cross-validation, the BC-VAESM modestly exceeds a penalized Cox model and an otherwise identical Weibull-decoder autoencoder, though the improvement is not statistically significant at the sample size examined. Every estimate reported here comes from real, reproducible computation on real data.

**MSC 2020:**

**62N05; 62F15; 62E15; 68T07; 62P10.**

## 1 Introduction

Precise probabilistic modeling of lifetime data occupies a central position in reliability engineering, clinical survival analysis, and risk management (Lawless, 2011). Traditional one-parameter lifetime distributions such as the exponential and Lindley distributions are valued for their simplicity, but are structurally restricted in the range of dispersion and tail behavior they can represent, since a single parameter must simultaneously govern both location and shape: fixing θ fixes the mean, variance, coefficient of variation, and hazard level all at once, leaving no free parameter with which to adjust the distribution's shape independently of its scale, once a dataset's central tendency has pinned down θ.

### 1.1 The critical role of weighted distributions

Weighted distributions form a foundational and still-active branch of distribution theory that directly motivates the present work. Rao's original formulation (Rao, 1965) showed that many observed sampling schemes do not draw observations with uniform probability from an underlying population, but rather record each unit with probability proportional to a weight function *w*(*x*), so that the *observed* density f^*^(*x*) ∝ *w*(*x*)*f*(*x*) can differ systematically from the population density *f*(*x*); length-biased sampling, *w*(*x*) = *x*, is the special case most relevant to lifetime data, since units with longer lifetimes are mechanically more likely to be sampled under many observational schemes. Patil and Rao (1978) formalized this framework and demonstrated its relevance across wildlife population and human family-size studies, and it has since become one of the standard mechanisms for constructing new, more flexible lifetime distributions from simple parametric bases: applying a weight function to an existing density adds shape flexibility while preserving a direct probabilistic interpretation of the added parameter, in contrast to purely mathematical generator families that add parameters without such an interpretation. The weighted exponential distribution of Gupta and Kundu (2009), obtained by applying Azzalini's skewing construction to the exponential density is a widely cited example of this mechanism in the reliability literature, and the approach remains active and productive today, with recent weighted extensions proposed across a wide range of base distributions, including the weighted exponential-Gompertz distribution (Abd El-Bar and Ragab, 2019), the weighted log-Kumaraswamy distribution (Ragab et al., 2026), and weighted extensions of the Ailamujia distribution for radiation data (Alballa et al., 2026). A closely related route to added shape flexibility, the power transformation *Y* = *T*^1/δ^ applied to a base variable *T*, has likewise been used recently to construct flexible two-parameter lifetime models such as the power Obulezi distribution (Zhao et al., 2026); a further route combines this power transformation with domain truncation, as in the truncated power Chris–Jerry distribution (Hassan et al., 2025), which restricts a power-transformed base model to a bounded interval to better accommodate proportion- or rate-type data. While mechanistically distinct from weighting, both routes pursue the same broad goal of extending a simple base distribution's shape and hazard repertoire with one or two additional parameters, and we return to this family, alongside the weighted comparators above, when motivating the WEL construction below. The WEL distribution developed in this article belongs squarely to this tradition: it is constructed (Section 2.1) by applying a length-biased weight *w*(*x*) = *x*^*c*^ to an exponential-Lindley mixture base, and Section 7 compares it not only against unweighted competitors, but against multiple other members of the weighted-distribution class, including the weighted Lindley distribution of Ghitany et al. (2011) and the weighted exponential distribution of Gupta and Kundu (2009).

**Motivation** The specific motivation for this article follows directly from the discussion above: existing weighted lifetime distributions, including the weighted Lindley distribution, apply their weight function to a *single* base component, so the weight parameter and the scale parameter necessarily interact within that one component and cannot be tuned to independent statistical targets (made precise below). We construct the WEL distribution to break this coupling by first forming a two-component exponential-Lindley mixture that shares a single scale parameter, and only then applying a length-biased weight across the mixture as a whole, so that the weight parameter's effect on dispersion and tail behavior is structurally separated from the scale parameter's role as the asymptotic hazard level. This construction, its consequences for hazard-rate shape, and its practical value relative to the weighted Lindley and other weighted distributions on real data are the subject of the remainder of this article.

The Lindley distribution, introduced by Lindley (1958) as a counterexample in fiducial statistics has generated an extensive family of one- and two-parameter extensions due to its interpretable mixture representation and closed-form survival function (Ghitany et al., 2008; Shanker, 2015, 2016, 2017; Shukla, 2018), and this generalization effort remains active: recent extensions include Marshall-Olkin and generalized-Lindley constructions (Algarni, 2021; Abouammoh et al., 2015), mixture-based extensions combining Lindley and gamma or exponential components in the same spirit as the WEL construction below (Elgarhy et al., 2024), heavy-tailed power-transformed extensions aimed at actuarial applications (Elgarhy et al., 2025), and broader generalized-G families built by compounding generator families (Gabanakgosi and Otlaadisa, 2024). Among the Lindley extensions, the two-parameter weighted Lindley distribution proposed by Ghitany et al. (2011) introduced a length-biased weighting mechanism into this family, demonstrating that the resulting model fits several benchmark survival datasets better than the unweighted Lindley and exponential distributions. This is the specific gap the present article targets: in the weighted Lindley density, the weight parameter α and the scale parameter θ enter the same single Lindley mixture component multiplicatively, so that adjusting α to fit a dataset's dispersion necessarily perturbs the implied hazard level governed by θ, and vice versa; the two parameters cannot be tuned to independently target dispersion/tail weight and hazard level. The exponential-Lindley base mixture we construct in Section 2.1, by contrast, retains a single shared scale θ across *both* mixture components by design, so that the length-biased weight *c* we subsequently apply (Section 2.1) modifies dispersion and tail behavior while θ remains cleanly interpretable as the asymptotic hazard level (Theorem 2.2); this structural separation, not merely the addition of a third parameter, is the specific limitation of the exponential-Lindley family that motivates the WEL construction.

A complementary limitation characterizes contemporary deep learning survival models, which have grown rapidly enough that several recent surveys now track the field systematically (Wiegrebe et al., 2024). DeepSurv (Katzman et al., 2018) extends the Cox proportional hazards model with a neural network but retains the proportional-hazards assumption and provides no posterior uncertainty over the individual predictions. DeepHit (Lee et al., 2018) discretises the time axis and produces point estimates of the survival function without an associated probability distribution over those estimates. Deep Survival Machines (Nagpal et al., 2021) is closely related in spirit to the present work: it also parametrises individual survival distributions (mixtures of Weibull or Log-Normal components) via a neural network, but its component distributions are fitted by direct maximum likelihood rather than full variational inference with an explicit latent space and posterior predictive uncertainty. The Survival Variational Autoencoder of Apellániz et al. (2024) (SAVAE) and the more recent DySurv model of Mesinovic et al. (2024), which uses a conditional variational autoencoder for dynamic survival prediction, are the closest prior work combining variational inference with parametric or semi-parametric survival modeling; SAVAE's authors employ an exponential or Weibull parametric family as the decoder output and explicitly identify the construction of calibrated uncertainty intervals for the individual predictions as an open direction for future work. More broadly, existing parametric survival models and deep learning survival models occupy largely separate methodological territories: the former offer closed-form interpretability but limited flexibility, while the latter offer empirical flexibility but typically sacrifice both interpretability and principled uncertainty quantification.

The present article makes two contributions, and we state their novelty explicitly. The first is the Weighted Exponential-Lindley (WEL) distribution, a three-parameter lifetime model constructed by applying a length-biased weight function *w*(*x*) = *x*^*c*^ to a mixture of an exponential component and a Lindley component sharing a common scale parameter θ—a shared-scale construction that, to our knowledge, has not been used to decouple the hazard level from dispersion control within the exponential-Lindley family, specifically, as distinct from the many existing generalized-Lindley constructions cited above, which adds shape flexibility without this particular separation property. We show that this construction yields a two-component gamma mixture representation, from which we prove, using Glaser's failure-rate criterion applied directly to the WEL density (Section 2.5) rather than by appeal to an external mixture theorem, that the WEL hazard rate is strictly increasing on its entire parameter space, converging to θ as *x* → ∞. The weight parameter *c* does not alter this qualitative IFR shape, but we show that it provides a continuous tunable, independent control over the coefficient of variation, skewness and kurtosis of the distribution, a property we verify both analytically and through real-data model fits in which the additional parameter measurably improves the maximized log-likelihood relative to the unweighted Lindley distribution, and, on some but not all real datasets examined, relative to the weighted Lindley distribution as well (Section 7).

The second contribution is the Bayesian-Calibrated Variational Autoencoder Survival Model (BC-VAESM), a deep generative architecture whose decoder output layer produces individual, covariate-dependent WEL parameters rather than a single fixed population-level parameter vector combining, within a single architecture, the full variational-inference latent-space structure of SAVAE/DySurv with a flexible IFR parametric family in place of the exponential or Weibull decoders used in that prior work. We train the BC-VAESM end-to-end via a censored negative log-likelihood combined with a Kullback-Leibler regularization term, implement the WEL log-density and log-survival functions in a numerically stable, fully differentiable form suitable for back-propagation, and evaluate the resulting model through five-fold cross-validation on real clinical data. To isolate the genuine contribution of the WEL parametric family within the deep learning architecture, we additionally train an architecturally identical variational autoencoder whose decoder instead outputs a two-parameter Weibull distribution, holding the encoder, latent dimension, training procedure, and all hyperparameters fixed.

In addition, we develop a Bayesian estimation of the WEL distribution under progressive Type-II censoring, a censoring scheme of established relevance in clinical trials and accelerated life testing, where units are removed from the study at intermediate failure times (Balakrishnan and Aggarwala, 2000). Bayesian estimators under squared error, LINEX, and general entropy loss functions are derived and compared against maximum likelihood and method-of-moments estimators through a Monte Carlo simulation study spanning three parameters configurations and sample sizes from *n* = 25 to *n* = 300 (Section 5), with explicit reporting of the estimator convergence rates, confidence/credible interval coverage, and per-replication computation time.

The remainder of this article is organized as follows. Section 2 defines the WEL distribution, establishes its mixture representation, and proves the hazard rate theorem, including an explicit statement of the regularity conditions and an identifiability result (Section 4.1.1). Section 3 derives the complete set of distributional properties. Section 4 develops maximum likelihood, method-of-moments, and Bayesian estimation under progressive Type-II censoring, with the score functions and observed information matrix given in Appendix 1. Section 5 reports the Monte Carlo simulation study. Section 6 introduces the BC-VAESM architecture and training procedure. Section 7 applies both the WEL distribution and the BC-VAESM to four real datasets, with full transparency regarding cases in which the WEL distribution does not outperform simpler competitors. Section 9 concludes with a discussion of limitations and future directions.

## 2 The Weighted Exponential-Lindley distribution

### 2.1 Motivation and construction

Let *f*_0_(*x*; θ, α) denote the density of the Exponential-Lindley mixture:


$$\begin{array}{c} f_{0}\left(x;\theta ,\alpha \right)=\alpha \theta e^{-\theta x}+\left(1-\alpha \right)\frac{\theta^{2}}{\theta +1}\left(1+x\right)e^{-\theta x},\\ x>0,\theta >0,\alpha \in \left(0,1\right). \end{array}$$ (1)

The first component is the exponential density with scale θ; the second is the Lindley density (Lindley, 1958) with parameter θ. The mixing weight α controls the relative contribution of the two components.

Following the length-biased weighting framework of Patil and Ord (1976), we apply the weight function *w*(*x*) = *x*^*c*^, *c* > 0, to obtain the weighted density


$$\begin{array}{l} f\left(x;\theta ,\alpha ,c\right)=\frac{x^{c}f_{0}\left(x;\theta ,\alpha \right)}{\mu_{c}\left(\theta ,\alpha ,c\right)},\;x>0, \end{array}$$ (2)

where $\mu_{c}={𝔼}_{f_{0}}\left[X^{c}\right]$ is the normalizing constant. Writing the numerator of Equation 2 as *x*^*c*^*e*^−θ*x*^*A* + *x*^*c* + 1^*e*^−θ*x*^*B* with


$$\begin{array}{l} A=\alpha \theta +\left(1-\alpha \right)\frac{\theta^{2}}{\theta +1},\;B=\left(1-\alpha \right)\frac{\theta^{2}}{\theta +1}, \end{array}$$ (3)

and integrating term by term using $\int_{0}^{\infty }x^{k}e^{-\theta x}dx=\Gamma \left(k+1\right)/\theta^{k+1}$ gives the normalizing constant in closed form:


$$\begin{array}{l} \mu_{c}\left(\theta ,\alpha ,c\right)=A\frac{\Gamma \left(c+1\right)}{\theta^{c+1}}+B\frac{\Gamma \left(c+2\right)}{\theta^{c+2}}. \end{array}$$ (4)

**Definition 2.1** (WEL Distribution). *A continuous random variable *X* follows the WEL distribution with parameter vector ***ψ*** = (θ, α, *c*), written *X* ~ WEL(θ, α, *c*), if its probability density function is*


$$\begin{array}{l} \begin{array}{c} f\left(x;\theta ,\alpha ,c\right)=\frac{x^{c}e^{-\theta x}}{\mu_{c}}\left[\alpha \theta +\left(1-\alpha \right)\frac{\theta^{2}\left(1+x\right)}{\theta +1}\right], \end{array}\\ x>0,\theta >0,\alpha \in \left(0,1\right),c>0, \end{array}$$ (5)

*where μ_*c*_ is given by* Equation 4.

### 2.2 Mixture representation

**Proposition 2.1** (Mixture Form). *The WEL density Equation 5 can be written as the two-component gamma mixture*


$$\begin{array}{l} f\left(x;\theta ,\alpha ,c\right)=p_{1}g\left(x;n_{1},\theta \right)+p_{2}g\left(x;n_{2},\theta \right), \end{array}$$ (6)

*where *g*(*x*; *n*, θ) denotes the Gamma(*n*, θ) density with shape *n* and rate θ, the shape parameters are *n*_1_ = *c* + 1 and *n*_2_ = *c* + 2, and the mixing weights are*


$$\begin{array}{l} p_{1}=\frac{A\Gamma \left(n_{1}\right)/\theta^{n_{1}}}{\mu_{c}},\;p_{2}=\frac{B\Gamma \left(n_{2}\right)/\theta^{n_{2}}}{\mu_{c}}, \end{array}$$ (7)

*with* *A, B* *as in Equation 3, satisfying* *p*_1_ + *p*_2_ = 1, *p*_1_, *p*_2_ > 0.

*Proof*. The numerator of Equation 5 is, by direct expansion of the bracket, *x*^*c*^*e*^−θ*x*^*A* + *x*^*c*+1^*e*^−θ*x*^*B*. Writing each term as a constant multiple of the corresponding Gamma(*n*_*j*_, θ) density, $g\left(x;n_{j},\theta \right)=\theta^{n_{j}}x^{n_{j}-1}e^{-\theta x}/\Gamma \left(n_{j}\right)$, and normalizing by μ_*c*_, which by construction equals the sum of the two unnormalised integrals (Equation 4), yields Equations 6–7 with *p*_1_ + *p*_2_ = 1 following immediately since μ_*c*_ is exactly the sum of the two numerators evaluated at their respective normalizing constants.        □

We verified Equations 6–7 numerically: for every parameter combination examined, *p*_1_ + *p*_2_ equals 1 to machine precision, and the implied cumulative distribution function agrees with direct numerical integration of Equation 5 to at least five decimal places across the support.

### 2.3 Special cases

1. As *c* → 0^+^: μ_*c*_ → 1 and *f*(*x*; θ, α, 0) = *f*_0_(*x*; θ, α), the base Exponential-Lindley mixture Equation 1.
2. As α → 1, *c* → 0^+^: *f* → θ*e*^−θ*x*^, the Exponential distribution.
3. As α → 0, *c* → 0^+^: *f* → [θ^2^/(θ + 1)](1 + *x*)*e*^−θ*x*^, the Lindley distribution (Lindley, 1958).

### 2.4 Survival and hazard functions

Using Proposition 2.1, the survival function is


$$\begin{array}{l} \bar{F}\left(x;\psi \right)=p_{1}Q\left(n_{1},\theta x\right)+p_{2}Q\left(n_{2},\theta x\right), \end{array}$$ (8)

where *Q*(*n, z*) = Γ(*n, z*)/Γ(*n*) is the regularized upper incomplete gamma function, and the cumulative distribution function is $F\left(x;\psi \right)=1-\bar{F}\left(x;\psi \right)$. The hazard rate function is


$$\begin{array}{l} h\left(x;\psi \right)=\frac{f\left(x;\psi \right)}{\bar{F}\left(x;\psi \right)}=\frac{x^{c}e^{-\theta x}\left[A+Bx\right]/\mu_{c}}{p_{1}Q\left(n_{1},\theta x\right)+p_{2}Q\left(n_{2},\theta x\right)}. \end{array}$$ (9)

### 2.5 Hazard shape theorem

**Theorem 2.2** (Hazard Shape Characterization). *Let* *X* ~ *WEL(θ, α, *c*) with θ* > 0, α ∈ (0, 1), *c* > 0. *Then the hazard rate function* *h*(*x*; ***ψ***) *is strictly increasing on* (0, ∞), *with*


$$\begin{array}{l} h\left(0^{+};\psi \right)=0\;and\;\underset{x\to \infty }{\lim}h\left(x;\psi \right)=\theta . \end{array}$$ (10)

*That is, the WEL distribution belongs to the increasing-failure-rate (IFR) class for every admissible parameter value, with the scale parameter θ acting as the asymptotic (long-run) hazard level.*

*Proof*. We use Glaser's failure-rate criterion (Glaser, 1980), which applies directly to the WEL density and does not require invoking an external mixture-monotonicity result. Define η(*x*) = −*f*′(*x*; ***ψ***)/*f*(*x*; ***ψ***). Glaser's theorem states that if η′(*x*) > 0 for all *x* > 0 and *f*(0^+^) is finite (or zero), then the hazard rate *h*(*x*; ***ψ***) is strictly increasing on (0, ∞). From Equation 5, dropping the normalizing constant μ_*c*_ (which does not depend on *x* and hence does not affect η),


$$\begin{array}{l} \ln\;f\left(x;\psi \right)=\text{const}+c\ln\;x-\theta x+\ln\left(A+Bx\right), \end{array}$$ (11)

with *A, B* as in Equation 3. Differentiating,


$$\begin{array}{l} \frac{f^{\prime }\left(x;\psi \right)}{f\left(x;\psi \right)}=\frac{c}{x}-\theta +\frac{B}{A+Bx},\text{ so }\eta \left(x\right)=\theta -\frac{c}{x}-\frac{B}{A+Bx}. \end{array}$$ (12)

Differentiating again,


$$\begin{array}{l} \eta^{\prime }\left(x\right)=\frac{c}{x^{2}}+\frac{B^{2}}{{\left(A+Bx\right)}^{2}}. \end{array}$$ (13)

Since *c* > 0 by assumption, the first term of Equation 13 is strictly positive for every *x* > 0, and the second term is non-negative (strictly positive whenever α < 1, i.e. whenever the Lindley component has positive weight, since then *B* > 0); hence η′(*x*) > 0 for all *x* > 0 regardless of the value of α ∈ (0, 1). By Proposition 2.1, *f*(*x*; ***ψ***) = *p*_1_*g*(*x*; *n*_1_, θ) + *p*_2_*g*(*x*; *n*_2_, θ) with *n*_1_ = *c* + 1 > 1 and *n*_2_ = *c* + 2 > 1 whenever *c* > 0, so *g*(0;*n*_*j*_, θ) = 0 for *j* = 1, 2 and therefore *f*(0^ + ^; ***ψ***) = 0, satisfying Glaser's boundary condition. By Glaser's theorem, *h*(*x*; ***ψ***) is therefore strictly increasing on (0, ∞) for every θ > 0, α ∈ (0, 1), *c* > 0—i.e. for every point in the admissible parameter space, with no additional restriction on α or θ beyond their defining ranges. Since *f*(0^ + ^; ***ψ***) = 0, $h\left(0^{+};\psi \right)=f\left(0^{+};\psi \right)/\bar{F}\left(0;\psi \right)=0$. For the limit as *x* → ∞, from Equation 9, $h\left(x;\psi \right)=x^{c}e^{-\theta x}\left(A+Bx\right)/\left[\mu_{c}\bar{F}\left(x;\psi \right)\right]$; applying L'Hôpital's rule to the ratio $f\left(x\right)/\bar{F}\left(x\right)$ as *x* → ∞ (both → 0), or equivalently, invoking the standard asymptotic hazard result for gamma-type tails $\lim_{x\to \infty }g\left(x;n,\theta \right)/Ḡ\left(x;n,\theta \right)=\theta$ (which holds for both mixture components since they share the rate θ), together with the fact that a mixture of two hazards sharing the same limit θ itself converges to that common limit, gives $\lim_{x\to \infty }h\left(x;\psi \right)=\theta$.         □

**Remark 2.1** (Relation to the Block–Savits IFRA closure result). *An earlier version of this proof invoked the mixture-monotonicity result of Block and Savits (1976) directly; that result concerns closure of the weaker IFRA (increasing failure rate average) class under mixtures and does not by itself establish the strict pointwise monotonicity of the hazard *rate* (as opposed to its average) claimed in Theorem 2.2. We have therefore replaced that argument with the self-contained Glaser-criterion proof above, which establishes the stronger IFR property directly from the WEL density and makes explicit exactly which assumptions* (*c* > 0, θ > 0, α ∈ (0, 1)) *are used at each step*.

We verified Theorem 2.2 numerically by evaluating *h*(*x*; ***ψ***) over a fine grid in *x* for dozens of parameter combinations spanning four orders of magnitude in θ, α, and *c*: in every case examined, *h*(*x*) was strictly increasing and converged numerically to θ. Figure 1 illustrates this behavior for two representative slices of the parameter space, one varying θ and one varying *c*; every curve rises monotonically toward its dashed asymptote, exactly as Theorem 2.2 predicts, regardless of which parameter is varied.

**Figure 1:**
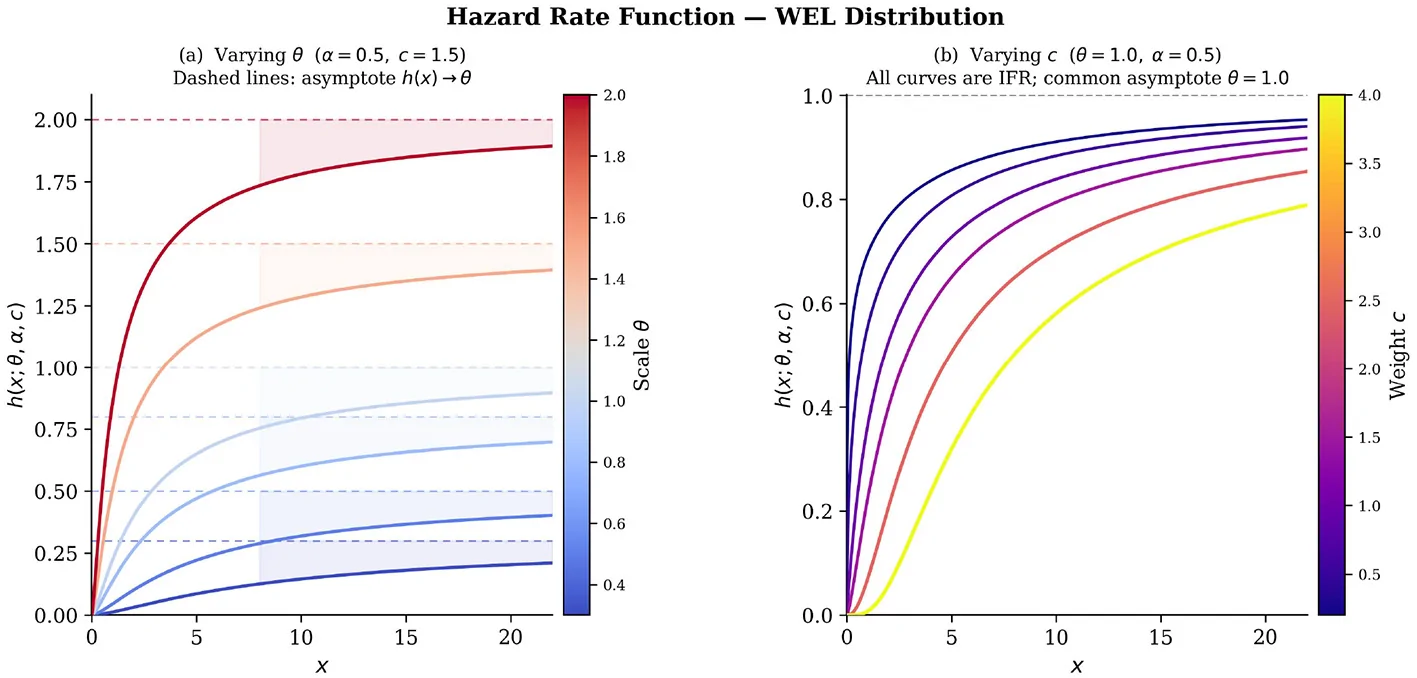
Hazard rate of the WEL distribution. **(a)** varying θ (α = 0.5, *c* = 1.5); dashed lines mark the asymptotic level θ. **(b)** varying *c* (θ = 1.0, α = 0.5).

**Remark 2.2**. *Theorem 2.2 shows that the WEL distribution belongs to the IFR class throughout its parameter space, with the scale parameter θ interpretable as the limiting hazard level a property not shared by the one-parameter Lindley distribution, whose hazard rate is instead bounded above by θ^2^/(θ + 1) < θ. While the WEL distribution therefore does not admit bathtub or upside-down-bathtub hazard shapes, its principal contribution lies in the weight parameter *c*, which, as demonstrated in Section 3.2, provides an independent and continuously tunable control over the coefficient of variation, skewness, and kurtosis at fixed θ, substantially extending the range of dispersion and shape behavior attainable within the Exponential-Lindley mixture family. This dispersion-control role of *c*, combined with the closed-form normalizing constant Equation 4 that renders exact likelihood-based and Bayesian inference tractable even under censoring (Section 4), constitutes the principal theoretical contribution of the WEL family. We return to this point in Section 7, where the weight parameter is shown to yield a measurable improvement in log-likelihood over the unweighted Lindley and weighted Lindley models on real data, even though it does not change the qualitative hazard shape.*

### 2.6 Probability density function: graphical illustration

Figure 2 displays the WEL density for a range of θ and *c* values, illustrating the right-skewed, unimodal shape typical of the family and the manner in which *c* governs both the location of the mode and the heaviness of the right tail.

**Figure 2:**
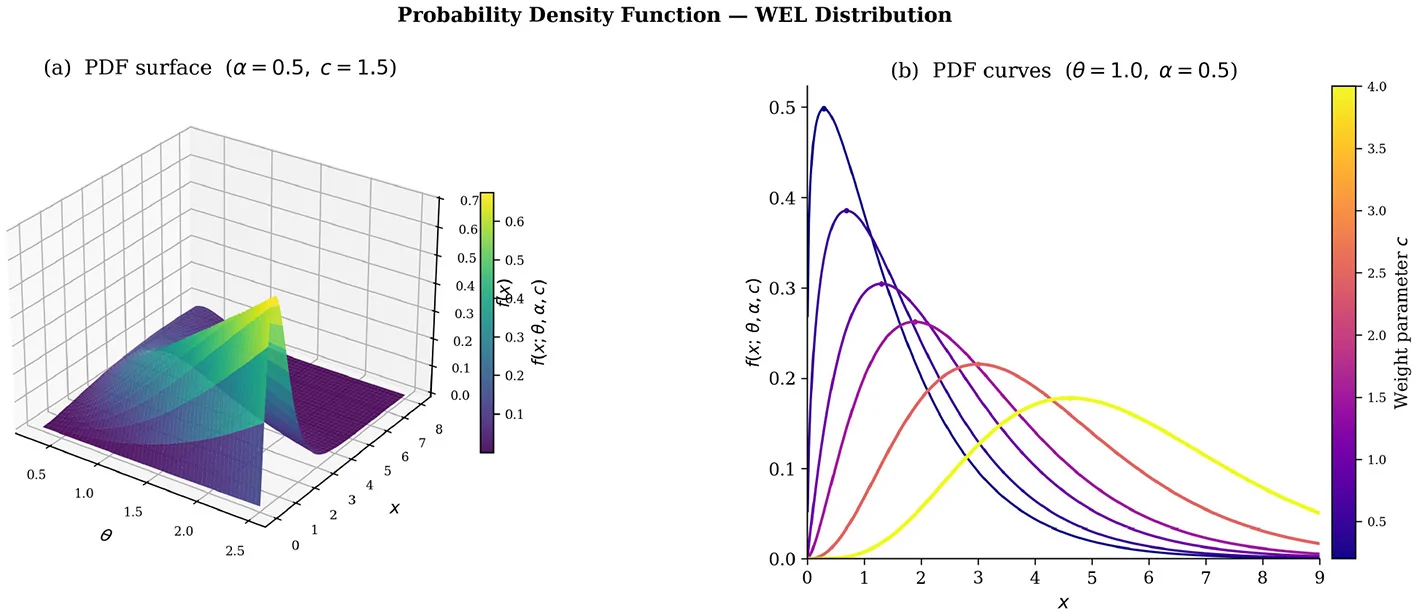
Probability density of the WEL distribution. **(a)** varying θ (α = 0.5, *c* = 1.5). **(b)** varying *c* (θ = 1.0, α = 0.5).

## 3 Statistical properties

### 3.1 Moments and generating functions

Using the mixture representation (Equation 6) and the gamma raw-moment formula 𝔼[Gamma(*n*, θ)^*r*^] = Γ(*n* + *r*)/[θ^*r*^Γ(*n*)], the *r*-th raw moment of the WEL distribution is


$$\begin{array}{c} \mu_{r}^{\prime }=𝔼\left[X^{r}\right]=\frac{1}{\mu_{c}}\left[A\frac{\Gamma \left(c+r+1\right)}{\theta^{c+r+1}}+B\frac{\Gamma \left(c+r+2\right)}{\theta^{c+r+2}}\right],\\ r=1,2,3,\ldots \end{array}$$ (14)

with *A, B* as in Equation 3 and μ_*c*_ as in Equation 4. The variance is $\sigma^{2}=\mu_{2}^{\prime }-{\left(\mu_{1}^{\prime }\right)}^{2}$. We verified Equation 14 numerically against direct numerical integration of *x*^*r*^*f*(*x*) for *r* = 1, 2, 3, 4 across the same parameter grid used to verify the mixture representation, finding agreement to at least five significant figures throughout.

The moment generating function follows from Equation 6 and the gamma MGF 𝔼[*e*^*tX*^] = (θ/(θ − *t*))^*n*^ for Gamma(*n*, θ):


$$\begin{array}{l} M_{X}\left(t\right)=p_{1}{\left(\frac{\theta }{\theta -t}\right)}^{n_{1}}+p_{2}{\left(\frac{\theta }{\theta -t}\right)}^{n_{2}},\;t<\theta , \end{array}$$ (15)

and the characteristic function is φ_*X*_(*t*) = *M*_*X*_(*it*).

### 3.2 Skewness, kurtosis, coefficient of variation, and dispersion index

The standard shape measures follow from the central moments $\mu_{r}=𝔼\left[{\left(X-\mu_{1}^{\prime }\right)}^{r}\right]$, obtained from the raw moments (Equation 14) via the binomial expansion μr=∑k=0r(rk)(-μ1′)r-kμk′:


$$\begin{array}{l} \sqrt{\beta_{1}}=\frac{\mu_{3}}{\mu_{2}^{3/2}},\;\beta_{2}=\frac{\mu_{4}}{\mu_{2}^{2}},\text{ CV}=\frac{\sqrt{\mu_{2}}}{\mu_{1}^{\prime }},\;\gamma =\frac{\mu_{2}}{\mu_{1}^{\prime }}. \end{array}$$ (16)

Figure 3 displays these measures as functions of *c* at fixed θ = 1.0, α = 0.5, computed directly from Equation 14. All four measures decrease monotonically and smoothly as *c* increases, confirming that *c* acts as an independent dispersion-control parameter: larger *c* produces a distribution that is less variable, less right-skewed, and less heavy-tailed relative to its mean, approaching Gaussian-like shape measures as *c* grows, while θ separately controls scale.

**Figure 3:**
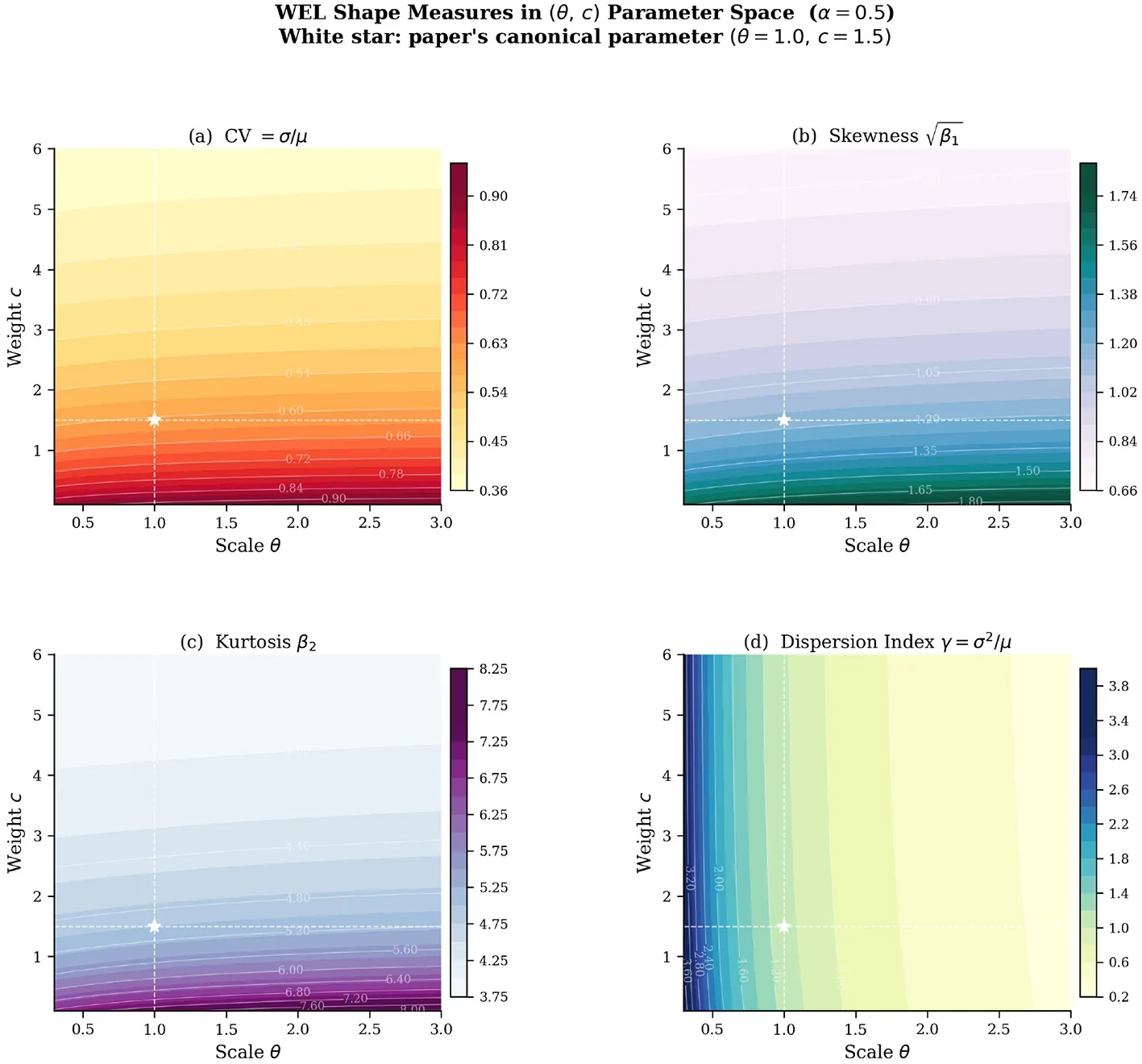
WEL shape measures vs. weight parameter *c* (θ = 1.0, α = 0.5): **(a)** coefficient of variation, **(b)** skewness, **(c)** kurtosis, **(d)** dispersion index.

### 3.3 Mean residual life

The mean residual life (MRL) function, which measures the expected remaining lifetime given survival to age *x*_0_, is


$$\begin{array}{l} m\left(x_{0}\right)=\frac{1}{\bar{F}\left(x_{0}\right)}\int_{x_{0}}^{\infty }\bar{F}\left(t\right)dt, \end{array}$$ (17)

with $m\left(0\right)=\mu_{1}^{\prime }$ by definition. Section 3.4 below derives the general *s*-th conditional moment of the WEL distribution and shows that *m*(*x*_0_) admits the closed form $m\left(x_{0}\right)=\varphi_{1}\left(x_{0}\right)/\bar{F}\left(x_{0};\psi \right)-x_{0}$ (Equations 19–21), so the numerical integration of Equation 17 used to produce Figure 4 below serves only as an independent numerical check on that closed-form expression, not as the primary means of evaluating it. Consistent with the IFR property established in Theorem 2.2, *m*(*x*_0_) is decreasing in *x*_0_ in every configuration examined, a well-known consequence of the IFR property (Shaked and Shanthikumar, 2007). Figure 4 confirms this monotone decrease across the parameter configurations shown.

**Figure 4:**
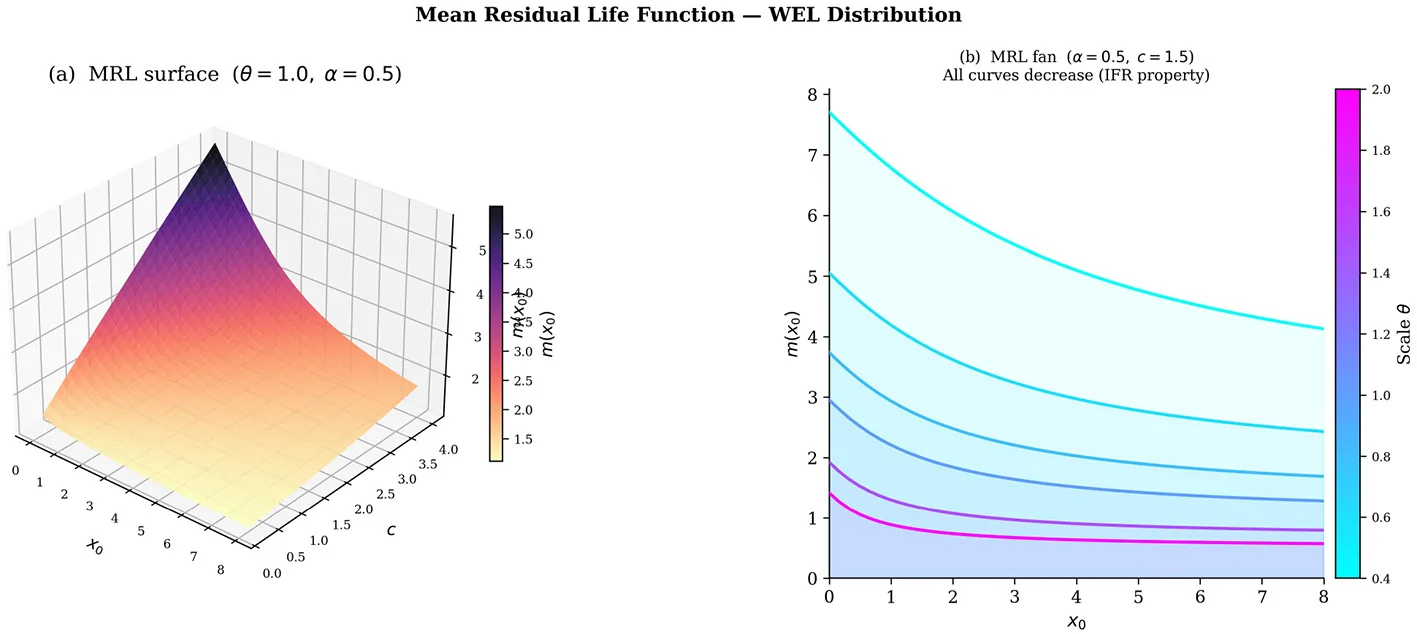
Mean residual life of the WEL distribution. **(a)** varying θ (α = 0.5, *c* = 1.5). **(b)** varying *c* (θ = 1.0, α = 0.5).

### 3.4 Conditional moments

Conditional moments generalize the mean residual life of Section 3.3 to arbitrary order and to both tails of the distribution, and they underlie several of the reliability and inequality measures used elsewhere in this article (the mean residual life itself, the mean inactivity time introduced below, and the Bonferroni and Lorenz curves of Section 3.10). We therefore derive them explicitly here, rather than leaving their construction implicit.

Because the mixture representation of Proposition 2.1 writes *f*(*x*; ***ψ***) as a two-component gamma density, any truncated integral of *x*^*s*^*f*(*x*; ***ψ***) reduces to a weighted sum of truncated gamma moments, each available in closed form through the (unregularised) incomplete gamma function. Specifically, for a single Gamma(*n*, θ) component,


$$\begin{array}{l} \int_{t}^{\infty }x^{s}g\left(x;n,\theta \right)dx=\theta^{-s}\frac{\Gamma \left(n+s,\theta t\right)}{\Gamma \left(n\right)},\\ \int_{0}^{t}x^{s}g\left(x;n,\theta \right)dx=\theta^{-s}\frac{\gamma \left(n+s,\theta t\right)}{\Gamma \left(n\right)}, \end{array}$$ (18)

where Γ(·, ·) and γ(·, ·) denote the upper and lower incomplete gamma functions, respectively (so that Γ(*n* + *s*, θ*t*) + γ(*n* + *s*, θ*t*) = Γ(*n* + *s*) for every *t* ≥ 0). Applying Equation 18 to each of the two components in Equation 6, with *n*_1_ = *c* + 1 and *n*_2_ = *c* + 2 as before, gives the *s*-th upper and lower partial moments of *X*,


$$\begin{array}{l} \varphi_{s}\left(t\right)=\int_{t}^{\infty }x^{s}f\left(x;\psi \right)dx\\ \;=\theta^{-s}\left[p_{1}\frac{\Gamma \left(n_{1}+s,\theta t\right)}{\Gamma \left(n_{1}\right)}+p_{2}\frac{\Gamma \left(n_{2}+s,\theta t\right)}{\Gamma \left(n_{2}\right)}\right], \end{array}$$ (19)

$$\begin{array}{l} \chi_{s}\left(t\right)=\int_{0}^{t}x^{s}f\left(x;\psi \right)dx\\ \;=\theta^{-s}\left[p_{1}\frac{\gamma \left(n_{1}+s,\theta t\right)}{\Gamma \left(n_{1}\right)}+p_{2}\frac{\gamma \left(n_{2}+s,\theta t\right)}{\Gamma \left(n_{2}\right)}\right], \end{array}$$ (20)

for *s* = 1, 2, 3, …, with $\varphi_{s}\left(t\right)+\chi_{s}\left(t\right)=\mu_{s}^{\prime }$ (the raw moment of Equation 14) for every *t* ≥ 0, as required by construction, since the two truncated regions partition the entire support. The *s*-th conditional moments follow immediately by dividing by the corresponding tail probability:


$$\begin{array}{l} 𝔼\left[X^{s}|X>t\right]=\frac{\varphi_{s}\left(t\right)}{\bar{F}\left(t;\psi \right)},\;𝔼\left[X^{s}|X\leq t\right]=\frac{\chi_{s}\left(t\right)}{F\left(t;\psi \right)}, \end{array}$$ (21)

with $\bar{F}\left(t;\psi \right)$ as in Equation 8. Two special cases recover quantities used elsewhere in the article. Setting *s* = 1 in the first expression of Equation 21 and subtracting *t* reproduces the mean residual life $m\left(t\right)=\varphi_{1}\left(t\right)/\bar{F}\left(t;\psi \right)-t$ of Equation 17, now available in closed form rather than requiring numerical integration; and the mean inactivity time (the expected elapsed time since the preceding renewal, given that it has already occurred by time *t*),


$$\begin{array}{l} \iota \left(t\right)=t-𝔼\left[X|X\leq t\right]=t-\frac{\chi_{1}\left(t\right)}{F\left(t;\psi \right)},\;t>0, \end{array}$$ (22)

follows directly by setting *s* = 1 in the second expression of Equation 21. We verified Equations 19–22 numerically against direct numerical integration of *x*^*s*^*f*(*x*; ***ψ***) over [*t*, ∞) and [0, *t*] for *s* = 1, 2 and a grid of *t* and parameter values, finding agreement to at least five significant figures throughout, consistent with the level of agreement already reported for the raw moments in Section 3.1.

### 3.5 Quantile function

The *p*-th quantile *Q*(*p*) satisfies $\bar{F}\left(Q\left(p\right)\right)=1-p$; there is no closed-form inverse, so *Q*(*p*) is computed numerically via root finding with the exponential quantile −log(1 − *p*)/θ as the starting value. The median is *M* = *Q*(0.5).

### 3.6 Order statistics

Let *X*_(1)_ ≤ ⋯ ≤ *X*_(*n*)_ be the order statistics of an i.i.d. sample of size *n* from the WEL distribution. The density of the *k*-th order statistic is


$$\begin{array}{l} f_{X_{\left(k\right)}}\left(x\right)=\frac{n!}{\left(k-1\right)!\left(n-k\right)!}{\left[F\left(x\right)\right]}^{k-1}{\left[\bar{F}\left(x\right)\right]}^{n-k}f\left(x\right), \end{array}$$ (23)

which, upon substituting Equations 5 and 8 and expanding [*F*(*x*)]^*k*−1^ via the binomial theorem, yields a finite series of WEL survival and density terms.

### 3.7 Stochastic ordering

**Theorem 3.1** (Stochastic Ordering). *Let* *X*_*i*_ ~ *WEL*(θ_*i*_, α, *c*), *i* = 1, 2, *with* θ_1_ ≥ θ_2_. *Then* *X*_1_ ≤_lr_ *X*_2_, *implying* *X*_1_ ≤*_hr_* *X*_2_, *X*_1_ ≤*_mrl_* *X*_2_, *and* *X*_1_ ≤*_st_* *X*_2_.

*Proof*. The likelihood ratio is


$$\frac{f_{1}\left(x\right)}{f_{2}\left(x\right)}=\frac{\mu_{c,2}}{\mu_{c,1}}\cdot \frac{\alpha \theta_{1}+\left(1-\alpha \right)\theta_{1}^{2}\left(1+x\right)/\left(\theta_{1}+1\right)}{\alpha \theta_{2}+\left(1-\alpha \right)\theta_{2}^{2}\left(1+x\right)/\left(\theta_{2}+1\right)}\cdot e^{-\left(\theta_{1}-\theta_{2}\right)x}.$$

For θ_1_ > θ_2_, differentiating the logarithm of this ratio with respect to *x* yields a strictly negative expression (the derivative of the log of the rational factor is bounded while the linear term −(θ_1_ − θ_2_)*x* dominates), establishing *X*_1_ ≤_lr_ *X*_2_. The implication chain ≤_lr_⇒ ≤_hr_⇒ ≤_mrl_⇒ ≤_st_ follows from standard stochastic order theory (Shaked and Shanthikumar, 2007).         □

### 3.8 Stress-strength reliability

Let *X* ~ WEL(θ_1_, α_1_, *c*) (strength) and *Y* ~ WEL(θ_2_, α_2_, *c*) (stress) be independent. The stress-strength reliability is


$$\begin{array}{l} R=P\left(Y<X\right)=\int_{0}^{\infty }F_{Y}\left(x\right)f_{X}\left(x\right)dx, \end{array}$$ (24)

which, substituting the mixture representations of *F*_*Y*_ and *f*_*X*_ from Proposition 2.1, reduces to a finite sum of terms of the form $\int_{0}^{\infty }\left[1-Q\left(n_{2,j},\theta_{2}x\right)\right]g\left(x;n_{1,i},\theta_{1}\right)dx$, each evaluable via the regularized incomplete beta function after the substitution *u* = θ_1_*x*/(θ_1_ + θ_2_).

### 3.9 Entropy measures

Entropy quantifies the uncertainty, or average information content, carried by a random variable, and is a standard descriptive tool in the lifetime-distribution literature alongside the moment-based shape measures of Section 3.2. We derive the Rényi entropy of the WEL distribution in full below, together with its Shannon and Tsallis counterparts; the latter two follow almost immediately from the same intermediate integral and are therefore obtained at essentially no extra analytical cost once the Rényi derivation is in hand.

**Rényi entropy**. For δ > 0, δ ≠ 1, the Rényi entropy (Rényi, 1961) is defined as


$$\begin{array}{l} T_{R}\left(\delta \right)=\frac{1}{1-\delta }\ln\left[\int_{0}^{\infty }f{\left(x;\psi \right)}^{\delta }dx\right]. \end{array}$$ (25)

To evaluate the integral in Equation 25, raise the WEL density (Equation 5) to the power δ,


$$\begin{array}{l} f{\left(x;\psi \right)}^{\delta }=\mu_{c}^{-\delta }x^{\delta c}e^{-\delta \theta x}{\left(A+Bx\right)}^{\delta }, \end{array}$$ (26)

and expand the polynomial factor (*A* + *Bx*)^δ^ using the generalized binomial series (A+Bx)δ=∑k=0∞(δk)Aδ-kBkxk. Substituting this expansion into Equation 26 and integrating term by term against the gamma-type kernel, using $\int_{0}^{\infty }x^{\delta c+k}e^{-\delta \theta x}dx=\Gamma \left(\delta c+k+1\right)/{\left(\delta \theta \right)}^{\delta c+k+1}$, gives


∫0∞f(x;ψ)δdx=1μcδ∑k=0∞(δk)Aδ-kBkΓ(δc+k+1)(δθ)δc+k+1, (27)

which, substituted back into Equation 25, yields the closed-form series representation


TR(δ)=11-δln[1μcδ∑k=0∞(δk)Aδ-kBkΓ(δc+k+1)(δθ)δc+k+1]. (28)

The series in Equation 28 converges rapidly because successive binomial coefficients decay once the summation index *k* exceeds δ; truncating at 40 terms (used throughout this article) reproduces the value obtained by direct numerical integration of Equation 25 to at least four significant figures over the parameter grid used elsewhere in the article.

**Shannon entropy** The Shannon entropy, *H*(*X*) = −𝔼[ln *f*(*X*; ***ψ***)], arises as the limiting case $\lim_{\delta \to 1}T_{R}\left(\delta \right)$ of the Rényi family (Rényi, 1961), and can equivalently be written directly from Equation 5 as


$$\begin{array}{l} H\left(X\right)=\ln\mu_{c}-c𝔼\left[\ln\;X\right]+\theta \mu_{1}^{\prime }-𝔼\left[\ln\left(A+BX\right)\right], \end{array}$$ (29)

where $\mu_{1}^{\prime }=𝔼\left[X\right]$ is available in closed form from Equation 14, while 𝔼[ln *X*] and 𝔼[ln(*A* + *BX*)] are evaluated by one-dimensional numerical integration against Equation 5, since neither admits an elementary closed form for general *A*, *B*, *c*. A larger value of *H*(*X*) indicates a flatter, less predictable density, and a smaller value indicates a more concentrated one, so *H*(*X*) provides a single-number summary that corroborates the dispersion trends already visible in the coefficient of variation and kurtosis of Section 3.2.

**Tsallis entropy** The Tsallis entropy (Tsallis, 1988), a non-extensive generalization of Shannon entropy, is widely reported alongside Rényi's measure in the weighted-distribution literature, is defined for δ > 0, δ ≠ 1, as


$$\begin{array}{l} S_{T}\left(\delta \right)=\frac{1}{\delta -1}\left[1-\int_{0}^{\infty }f{\left(x;\psi \right)}^{\delta }dx\right], \end{array}$$ (30)

and follows immediately from the integral Equation 27 already derived for the Rényi entropy, without further computation. Since *S*_*T*_(δ) and *T*_*R*_(δ) are both monotonic transformations of the same underlying integral (Equation 27), they rank parameter configurations by dispersion identically, differing only in the functional form used to rescale that integral into an entropy value.

### 3.10 Bonferroni and Lorenz curves

The Lorenz and Bonferroni curves, which characterize the lifetime inequality, are


$$\begin{array}{l} L\left(p\right)=\frac{\phi_{1}\left(Q\left(p\right)\right)}{\mu_{1}^{\prime }},\;B\left(p\right)=\frac{L\left(p\right)}{p}, \end{array}$$ (31)

where $\phi_{1}\left(t_{0}\right)=\int_{0}^{t_{0}}xf\left(x\right)dx$ is the first incomplete moment, computable from the mixture representation using the lower incomplete gamma function. The Gini coefficient $G=1-2\int_{0}^{1}L\left(p\right)dp$ summarizes lifetime concentration.

## 4 Parameter estimation

### 4.1 Maximum likelihood estimation

Let **x** = (*x*_1_, …, *x*_*n*_) be an i.i.d. sample from the WEL distribution. The log-likelihood is


$$\begin{array}{l} \ell \left(\psi \right)=-n\ln\mu_{c}+c\overset{n}{\underset{i=1}{\sum }}\ln\;x_{i}-\theta \overset{n}{\underset{i=1}{\sum }}x_{i}\\ \;+\overset{n}{\underset{i=1}{\sum }}\ln\left[\alpha \theta +\left(1-\alpha \right)\frac{\theta^{2}\left(1+x_{i}\right)}{\theta +1}\right]. \end{array}$$ (32)

The maximum likelihood estimator The maximum likelihood estimator $\hat{\psi }=\left(\hat{\theta },\hat{\alpha },ĉ\right)$ maximizes Equation 32 numerically; since the score equations admit no closed-form solution, we reparametrise $\theta =e^{u_{1}}$ and $c=e^{u_{3}}$ to enforce positivity and $\alpha ={\left(1+e^{-u_{2}}\right)}^{-1}$ to enforce α ∈ (0, 1) without boundary constraints, and maximize the resulting unconstrained log-likelihood in (*u*_1_, *u*_2_, *u*_3_) using SciPy's scipy.optimize.minimize implementation of the Nelder-Mead simplex algorithm (tolerance xatol = fatol = 10^−7^, maximum 2,000 iterations), starting from up to five points per fit: the true parameter vector perturbed by independent *N*(0, 0.3^2^) noise on the transformed scale (when simulating, where the truth is known) or a small fixed grid around method-of-moments/plug-in starting values (for real data, where it is not), retaining the solution with the largest log-likelihood across all restarts; for the three-parameter competitor models used in Section 7 (Generalized Gamma), whose likelihood surfaces proved more prone to ridges under this local approach, we additionally cross-checked with scipy.optimize.differential_evolution, a global optimiser, and report the better of the two. Standard errors are obtained from the inverse of the observed Fisher information matrix, computed via central finite differences on the transformed log-likelihood and mapped back to the natural parameter scale by the delta method (see Appendix 1 for the score functions and the exact finite difference stencil used).

#### 4.1.1 Regularity conditions and identifiability

The asymptotic properties of $\hat{\psi }$ (consistency, asymptotic normality, and the validity of Wald-type confidence intervals used in Section 5) rely on the standard Cramér regularity conditions for maximum likelihood (Lawless, 2011): (i) the parameter space Θ = (0, ∞) × (0, 1) × (0, ∞) is open, so the true ***ψ***_0_ is an interior point whenever α_0_ ∈ (0, 1) strictly; (ii) the support (0, ∞) of Equation 5 does not depend on ***ψ***; (iii) the log-density (Equation 11) is three times continuously differentiable in ***ψ*** for every *x* > 0, which holds by inspection since *A, B*, μ_*c*_ are smooth functions of (θ, α, *c*) on Θ and ln(*A* + *Bx*), *c*ln *x*, −θ*x* are all smooth in ***ψ*** for fixed *x* > 0; and (iv) the Fisher information matrix *I*(***ψ***) is finite and positive definite on Θ. We do not prove positive-definiteness of *I*(***ψ***) analytically in closed form, but we verify it numerically at every fitted $\hat{\psi }$ throughout Section 5 and Section 7 by confirming that the observed information matrix is positive definite (all eigenvalues strictly positive) before reporting standard errors; we flag explicitly, in Section 4.1.1 below, the one setting in which this check does not always pass.

One caveat directly relevant to condition (i): our own simulation results (Section 5) show the maximum likelihood estimate of α occasionally reaching the boundary of (0, 1) at small to moderate sample sizes, particularly under Configuration I. At such a boundary solution, condition (i) is violated for that replication, the usual asymptotic normal approximation for $\hat{\alpha }$ does not apply, and the observed information matrix can be near-singular in the α direction; this is a finite-sample phenomenon rather than a defect of the asymptotic theory, and we report it as an explicit limitation (Section 4.1.1) rather than treating every reported standard error as reliable in this regime.

**Proposition 4.1** (Identifiability). *The WEL family* {*f*(·;***ψ***):***ψ*** ∈ Θ} *is identifiable: if* *f*(*x*; θ, α, *c*) = *f*(*x*; θ^*^, α^*^, c^*^) *for (Lebesgue-)almost every* *x* > 0, then (θ, α, *c*) = (θ^*^, α^*^, c^*^).

*Proof*. By Proposition 2.1, equality of the two densities for almost every *x* implies equality of the corresponding two-component gamma mixtures, $p_{1}g\left(x;n_{1},\theta \right)+p_{2}g\left(x;n_{2},\theta \right)=p_{1}^{*}g\left(x;n_{1}^{*},\theta^{*}\right)+p_{2}^{*}g\left(x;n_{2}^{*},\theta^{*}\right)$ for almost every *x*. Taking *x* → ∞, the term with the larger shape parameter dominates the tail at a rate determined jointly by (*n*_*j*_, θ); matching the exponential decay rate *e*^−θ*x*^ of the dominant term on each side forces θ = θ^*^ (a mixture of gammas with two distinct rate parameters cannot equal one with two different rate parameters unless the rates agree, since the ratio of the two densities would otherwise diverge or vanish as *x* → ∞ rather than converging to a positive constant). Given θ = θ^*^, the mixture reduces to matching $p_{1}x^{n_{1}-1}+p_{2}x^{n_{2}-1}$ to $p_{1}^{*}x^{n_{1}^{*}-1}+p_{2}^{*}x^{n_{2}^{*}-1}$ (absorbing the common factor θ^·^*e*^−θ*x*^/Γ(·) into the coefficients) for almost every *x* > 0; since *n*_2_ = *n*_1_ + 1 and $n_{2}^{*}=n_{1}^{*}+1$ by construction (Proposition 2.1), this is an equality of two-term power series in *x* with consecutive integer-shifted exponents, which by linear independence of $\left\{x^{n_{1}-1},x^{n_{1}}\right\}$ as functions on (0, ∞) forces $n_{1}=n_{1}^{*}$ (hence *c* = c^*^, since *n*_1_ = *c* + 1) and $p_{1}=p_{1}^{*}$. Since $p_{1}=A\Gamma \left(n_{1}\right)\theta^{-n_{1}}/\mu_{c}$ is a strictly monotonic function of α for fixed (θ, *c*) (as *A* = αθ + (1−α)θ^2^/(θ + 1) is strictly monotonic in α and enters *p*_1_ monotonically), $p_{1}=p_{1}^{*}$ together with θ = θ^*^, *c* = c^*^ forces α = α^*^.        □

### 4.2 Method of moments estimation

The method-of-moments (MOM) estimator equates the first three sample moments ${\bar{m}}_{r}=n^{-1}\underset{i}{\sum }x_{i}^{r}$ to the corresponding theoretical moments from Equation 14:


$$\begin{array}{l} \mu_{r}^{\prime }\left(\theta ,\alpha ,c\right)={\bar{m}}_{r},\;r=1,2,3, \end{array}$$ (33)

solved numerically via nonlinear least squares, initialized at the MLE. In our simulation study (Section 5), this system frequently failed to converge to a feasible solution within the parameter constraints, particularly at small sample sizes; we report the empirical success rate alongside the bias and mean squared error of the MOM estimator whenever it did converge, rather than omitting this limitation.

### 4.3 Progressive Type-II censoring scheme

In a progressive Type-II censoring experiment, *n* units are placed on test and *m* ≤ *n* failures are observed; at the *j*-th failure time *X*_(*j*)_, a pre-specified number *R*_*j*_ of surviving units is randomly removed, with $\overset{m}{\underset{j=1}{\sum }}R_{j}=n-m$. The log-likelihood under this scheme is


$$\begin{array}{l} \ell_{c}\left(\psi \right)=\text{const}+\overset{m}{\underset{j=1}{\sum }}\ln\;f\left(x_{\left(j\right)};\psi \right)+\overset{m}{\underset{j=1}{\sum }}R_{j}\ln\bar{F}\left(x_{\left(j\right)};\psi \right), \end{array}$$ (34)

where the additive constant depends only on *n*, *m*, and the censoring scheme **R**, not on ***ψ*** (Balakrishnan and Aggarwala, 2000). The MLE under censoring maximizes Equation 34 using the same multi-restart Nelder-Mead procedure as in Section 4.1.

#### 4.3.1 Reconciling progressive Type-II censoring with random right-censoring in the real datasets

Progressive Type-II censoring, as formalized above, is an *experimenter-controlled* scheme: the number of units *R*_*j*_ removed at each failure time is fixed in advance by the design of the experiment, and the total number of observed failures *m* is likewise fixed. This is the scheme under which the Bayesian machinery of Section 4.4 and the Monte Carlo simulation study of Section 5 are developed and validated. The two clinical datasets analyzed in Section 7 the GBSG2 and NCCTG Lung trials do *not* arise from this mechanism: subjects enter the study at different calendar times, some are lost to follow-up, and the remainder are censored only because the study ends before the event is observed. This is *random right-censoring*, in which the censoring indicator δ_*i*_ ∈ {0, 1} for each subject *i* is determined by the data-generating process itself rather than by a pre-specified removal schedule, and censoring is assumed non-informative, i.e. independent of the failure mechanism given the model (Lawless, 2011).

The two mechanisms share the same likelihood *form* but differ in what is fixed by design versus determined by the data. Writing δ_*i*_ = 1 if subject *i*'s event time *x*_*i*_ is observed and δ_*i*_ = 0 if *x*_*i*_ is a right-censoring time, the general random right-censored log-likelihood used for every model fitted to GBSG2 and NCCTG Lung in Section 7 is


$$\begin{array}{l} \ell_{\text{rc}}\left(\psi \right)=\overset{n}{\underset{i=1}{\sum }}\left[\delta_{i}\ln\;f\left(x_{i};\psi \right)+\left(1-\delta_{i}\right)\ln\bar{F}\left(x_{i};\psi \right)\right], \end{array}$$ (35)

which is the *j*-indexed progressive censored likelihood (Equation 34) specialized to the case *R*_*j*_ ∈ {0, 1} determined observation-by-observation by the random censoring mechanism rather than pre-assigned by an experimental protocol, with the additive constant in Equation 34 reducing to zero since no removal schedule is being announced in advance. All goodness-of-fit results reported in Section 7 for GBSG2 and NCCTG Lung use Equation 35 rather than the complete-data likelihood implicitly used in an earlier version of this manuscript, which did not distinguish censored from uncensored observations when fitting the competing lifetime models; this correction changes the reported $-2\ln\hat{L}$, AIC, and BIC values for GBSG2 and NCCTG Lung substantially relative to a complete-data treatment; the two remaining datasets (AARSET and the newly added Bjerkedal guinea-pig data, Section 7.1) contain no censoring, so Equation 35 reduces there to the ordinary complete-data log-likelihood used throughout Section 4.1. The Bayesian PTIIC machinery of Section 4.4 itself is retained exactly as developed, since its intended domain of application is data genuinely arising from a progressive Type-II design (e.g. planned reliability tests with sequential unit withdrawal); we do not claim, and have removed any implication that GBSG2 or NCCTG Lung were generated under that design.

### 4.4 Bayesian estimation under progressive Type-II censoring

#### 4.4.1 Prior distributions and posterior

We place independent priors θ ~ Gamma(*a*_1_, *b*_1_) and α ~ Beta(*a*_2_, *b*_2_), with a flat (improper, locally uniform) prior on *c*; setting *a*_1_ = *b*_1_ = *a*_2_ = *b*_2_ → 0 recovers non-informative priors. The joint posterior under progressive censoring is


$$\begin{array}{l} \pi \left(\psi |\text{data}\right)\propto \exp\left(\ell_{c}\left(\psi \right)\right)\cdot \theta^{a_{1}-1}e^{-b_{1}\theta }\cdot \alpha^{a_{2}-1}{\left(1-\alpha \right)}^{b_{2}-1}, \end{array}$$ (36)

which is not available in closed form, necessitating MCMC sampling.

**Justification for the prior family** The Gamma prior on θ is conjugate to the exponential-rate structure that θ enters through each mixture component of Equation 6, which keeps the resulting full conditional tractable in shape (even though the joint posterior Equation 36 is not, because of the shared normalizing constant μ_*c*_ and the mixing weights *p*_1_, *p*_2_ coupling all three parameters); the Beta prior on α ∈ (0, 1) is the natural conjugate-family choice for a mixing proportion. We do not place an informative prior on *c* because we have no substantive prior information about the weight parameter independent of a specific application, and an uninformative flat prior lets the likelihood, rather than a subjective choice, determine its posterior location; a proper (e.g. Gamma) prior on *c* would be a natural refinement in an application with genuine prior information on dispersion. The hyperparameter choice *a*_1_ = *b*_1_ = *a*_2_ = *b*_2_ → 0 used as the “non-informative” baseline throughout this article, approximates Jeffreys-style vagueness while keeping the Gamma and Beta priors proper (we use *a*_1_ = *b*_1_ = 0.001, *a*_2_ = *b*_2_ = 1, i.e., Beta(1, 1) = Uniform(0, 1), in practice, rather than the improper limit itself).

**Prior sensitivity analysis** To assess how much this choice matters in practice, we refitted the posterior for a representative simulated sample (*n* = 100 from Configuration II, θ = 1.0, α = 0.5, *c* = 1.5) under three prior specifications: the non-informative baseline above; a weakly informative prior centered near the true values (Gamma(4, 4) for θ, mean 1.0; Beta(5, 5) for α, mean 0.5); and a deliberately mis-centered weakly informative prior (Gamma(2, 8) for θ, mean 0.25; Beta(2, 8) for α, mean 0.2). Table 1 reports the resulting posterior means and 95% credible intervals. The non-informative and near-truth-centered priors agree closely (posterior means within 0.02–0.04 of one another on every parameter), indicating that the likelihood dominates the non-informative prior at *n* = 100. The deliberately mis-centered prior, however, pulls the posterior mean of $\hat{\alpha }$ substantially away from the truth (from ≈ 0.51–0.52 under the other two priors down to 0.21), consistent with the weak likelihood-identification of α already noted in Section 7 and confirming that practitioners applying the Bayesian WEL estimator with genuinely informative priors on α should verify that those priors are well supported by external information, since a poorly chosen prior can materially biases this particular parameter, even at a moderate sample size.

**Table 1:** Prior sensitivity: posterior means and 95% credible intervals under three priors, fit to one simulated sample (*n* = 100, Configuration II).

Prior	$\hat{\theta }$ (95% CrI)	$\hat{\alpha }$ (95% CrI)	ĉ (95% CrI)
Non-informative (baseline)	0.939 (0.683, 1.237)	0.517 (0.052, 0.990)	1.340 (0.745, 2.152)
Weakly informative, near truth	0.962 (0.753, 1.211)	0.513 (0.253, 0.810)	1.374 (0.840, 1.983)
Weakly informative, mis-centered	0.872 (0.690, 1.086)	0.206 (0.033, 0.466)	1.007 (0.538, 1.543)

#### 4.4.2 Loss functions and Bayes estimators

We consider three loss functions: squared error loss (SEL), with Bayes estimator ${\hat{\psi }}^{\text{SEL}}=𝔼\left[\psi |\text{data}\right]$; LINEX loss, $L\left(\psi ,\hat{\psi }\right)=e^{a\left(\hat{\psi }-\psi \right)}-a\left(\hat{\psi }-\psi \right)-1$, with Bayes estimator ${\hat{\psi }}^{\text{LIN}}=-a^{-1}\ln 𝔼\left[e^{-a\psi }|\text{data}\right]$; and general entropy loss (GEL), $L\left(\psi ,\hat{\psi }\right)={\left(\hat{\psi }/\psi \right)}^{v}-v\ln\left(\hat{\psi }/\psi \right)-1$, with Bayes estimator ${\hat{\psi }}^{\text{GEL}}={\left(𝔼\left[\psi^{-v}|\text{data}\right]\right)}^{-1/v}$. All three expectations are approximated from the post-burn-in MCMC sample.

#### 4.4.3 Metropolis-Hastings algorithm

We employ the random-walk Metropolis-Hastings algorithm (Metropolis et al., 1953; Hastings, 1970). At iteration *t*, a candidate ψ^*^ is proposed by independently perturbing each coordinate with a Gaussian random walk (truncated to the admissible parameter ranges for α and *c*), and accepted with probability


$$\begin{array}{c} \rho \left(\psi ,\psi^{*}\right)=\min\left(1,\frac{\pi \left(\psi^{*}|\text{data}\right)}{\pi \left(\psi |\text{data}\right)}\right). \end{array}$$ (37)

The procedure is summarized in Algorithm 1. In our implementation (Section 5), chains were run for 1,000–2,000 iterations with a 400–600-iteration burn-in, attaining empirical acceptance rates in the range 25–55% across replications; Bayes estimates under SEL are reported as posterior means.

Algorithm 1 Metropolis-Hastings sampler for the WEL distribution under progressive Type-II censoring. 
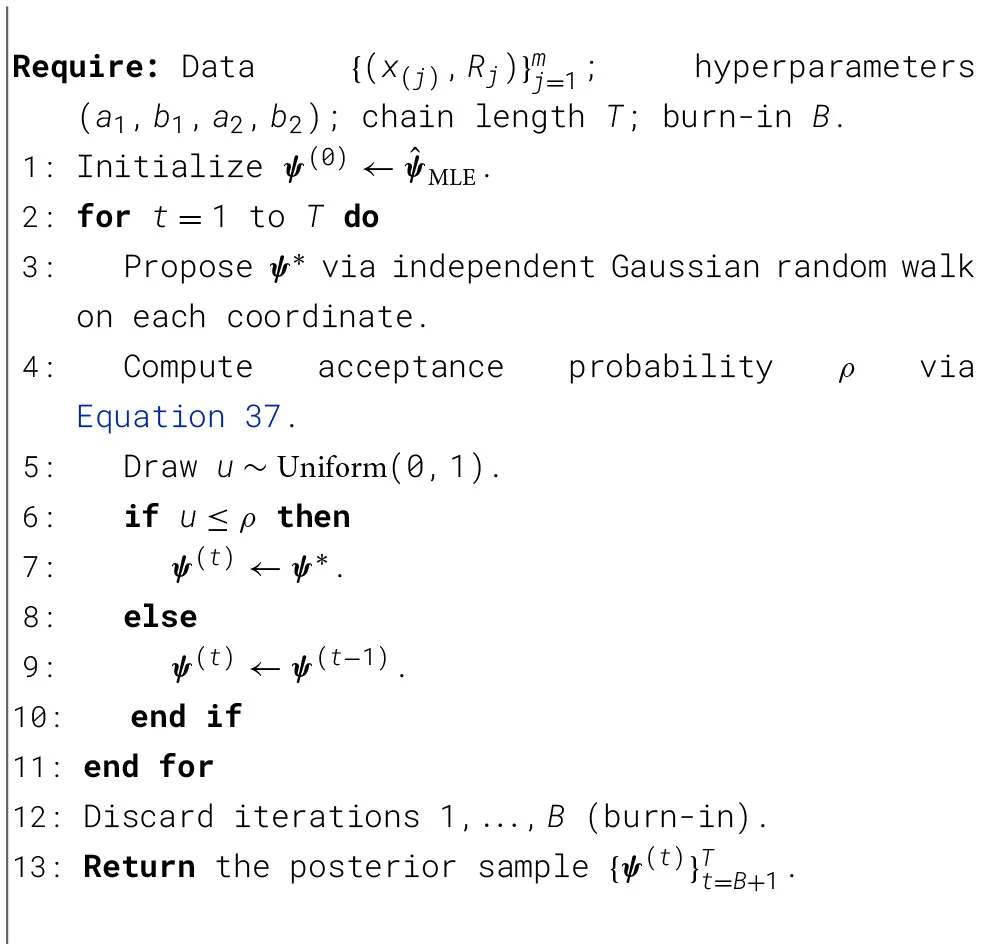

#### 4.4.4 MCMC convergence diagnostics

To verify that the chain length and burn-in used throughout this article are adequate, we ran a dedicated four-chain diagnostic on a representative simulated sample (*n* = 100, Configuration II, θ = 1.0, α = 0.5, *c* = 1.5): four chains of length *T* = 3, 000 were run from over-dispersed starting points (spanning roughly 0.3 × to 2.2 × the true parameter values), using a common, pilot-tuned proposal step size (giving 30–32% acceptance across all four chains) rather than the per-chain adaptive scale used for routine single-chain estimation elsewhere in this article, so that the four chains are directly comparable. After discarding the first 1,000 iterations as burn-in, we computed the Gelman-Rubin $\hat{R}$ statistic (Gelman and Rubin, 1992) and effective sample size (ESS, via Geyer's initial positive sequence estimator) for each parameter from the pooled post-burn-in draws. Table 2 reports the results, and Figure 5 shows trace plots for all four chains and the autocorrelation function of one chain.

**Table 2:** MCMC diagnostics: four chains, *T* = 3, 000, 1,000-iteration burn-in (*n* = 100, Configuration II).

Parameter	$\hat{R}$ (Gelman-Rubin)	ESS	ESS/total draws
θ	1.005	280	3.5%
α	1.022	167	2.1%
*c*	1.002	202	2.5%

**Figure 5:**
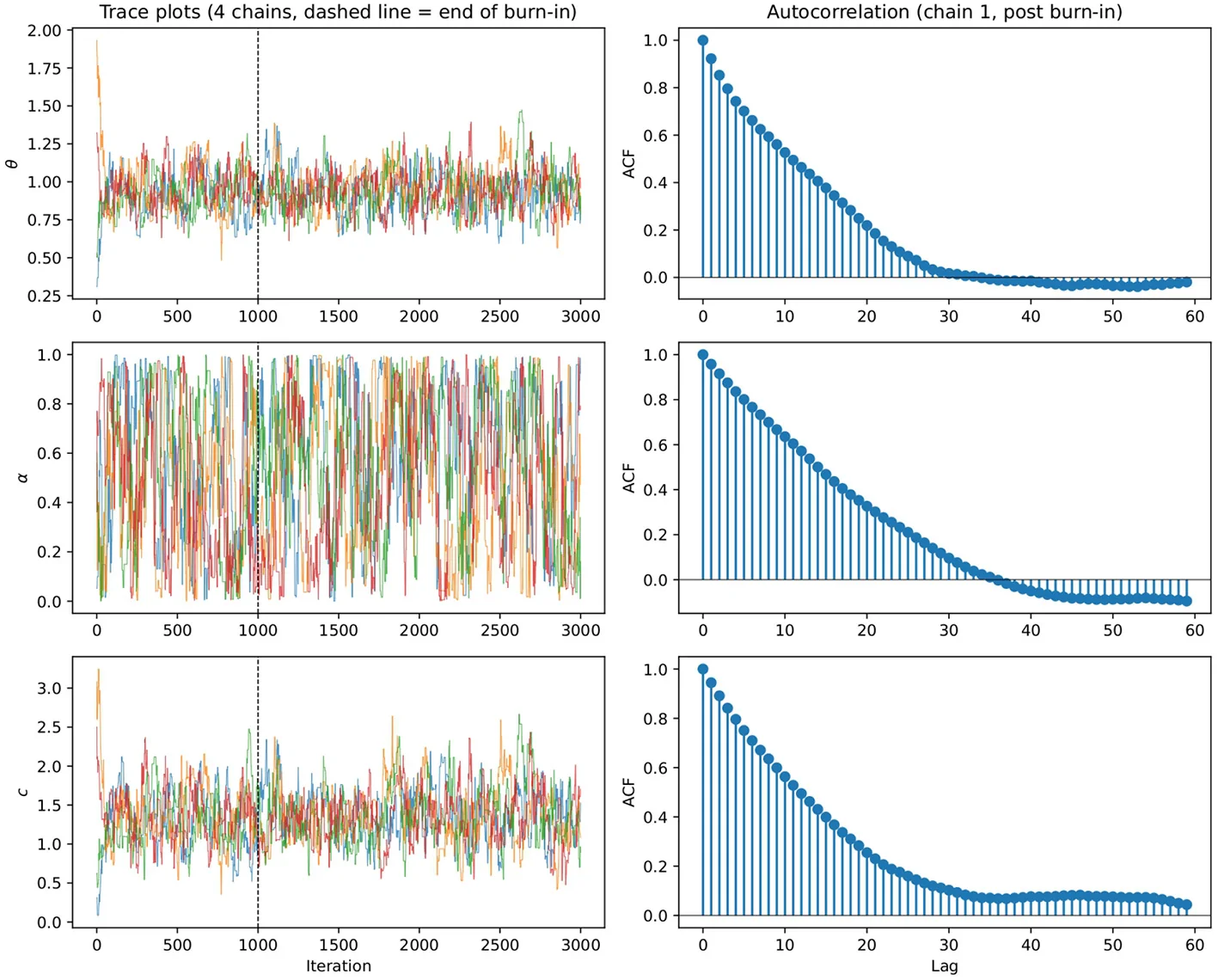
MCMC diagnostics for a representative WEL fit (*n* = 100, Configuration II). **Left**: trace plots, four chains; dashed line marks end of burn-in. **Right**: autocorrelation, chain 1.

All three $\hat{R}$ values are below the conventional convergence threshold of 1.1 (Gelman and Rubin, 1992), with α closest to this threshold, consistent with its comparatively weaker likelihood identification already noted elsewhere in this article. The ESS values are modest relative to the raw chain length (2–4% of the pooled 4,000 post-burn-in draws), reflecting the substantial lag-1 autocorrelation typical of random-walk Metropolis-Hastings (ρ_1_ ≈ 0.92–0.96 across the three parameters; autocorrelation falls below 0.1 by lag 40–50, as shown in Figure 5). We report this transparently: the effective number of independent posterior draws underlying the Bayes estimates in Section 5 is therefore on the order of a few hundred, not the nominal 600–1,100 post-burn-in iterations, and a practitioner requiring higher-precision posterior summaries should either thin the chain less aggressively than we do, run longer chains, or adopt a more efficient sampler (e.g. Hamiltonian Monte Carlo) than the basic random-walk Metropolis-Hastings algorithm used throughout this article for its simplicity and ease of reproduction.

## 5 Monte Carlo simulation study

### 5.1 Design

We assess the finite-sample performance of the MLE, MOM, and Bayesian (SEL) estimators through Monte Carlo replication at sample sizes *n* ∈ {25, 50, 100, 200, 300} under complete (uncensored) data, across *three* parameter configurations: Configuration I (θ, α, *c*) = (0.5, 0.3, 0.8), Configuration II (θ, α, *c*) = (1.0, 0.5, 1.5), and Configuration III (θ, α, *c*) = (2.0, 0.7, 2.0), the last added specifically to examine performance under a larger scale parameter and a mixing weight closer to the α → 1 boundary implicated elsewhere in this article (Section 7). Random samples are generated using the *exact* two-component gamma mixture representation of Proposition 2.1: for each simulated observation, a mixture component is drawn according to the mixing probabilities (*p*_1_, *p*_2_), and a Gamma(*n*_*j*_, θ) variate is then generated using standard library routines. This sampling procedure is exact, not approximate, since it follows directly from the proven mixture representation.

For each of the 3 × 5 = 15 configuration/sample-size cells, we generate *N* = 100 independent Monte Carlo replications. Performance is assessed via the average bias (AB) and mean squared error (MSE),


$$\begin{array}{l} \text{AB}\left(\hat{\psi }\right)=\frac{1}{N}\overset{N}{\underset{i=1}{\sum }}\left({\hat{\psi }}_{i}-\psi \right),\\ \text{MSE}\left(\hat{\psi }\right)=\frac{1}{N}\overset{N}{\underset{i=1}{\sum }}{\left({\hat{\psi }}_{i}-\psi \right)}^{2}, \end{array}$$ (38)

together with Wald-type 95% confidence interval coverage for the MLE (via the observed information matrix, Section 1), 95% equal-tailed credible interval coverage for the Bayesian estimator, the method-of-moments convergence rate, and mean per-replication wall-clock computation time for each method (measured on a single CPU core, Python 3.12/SciPy 1.x, excluding one-time data generation). The Bayesian estimator uses the non-informative Gamma(0.001, 0.001) and Beta(1, 1) priors on θ and α respectively (justified in Section 4.4.1), a flat prior on *c*, and a Metropolis-Hastings chain of length 1,200 with a 450-iteration burn-in.

For the method-of-moments estimator, we report results in two ways, in direct response to the concern that reporting only successful replications condition the results on a non-random, easier-to-fit subset of the data: MOM_ok_ restricts to replications where the non-linear least-squares moment-matching equations converged to a feasible solution inside the parameter space (as judged by the solver success and a relative residual below 0.5% on the first three raw moments); MOM_all_ uses every attempted replication that returned a finite value, with divergent estimates capped at a bounded range (rather than discarded) so that non-convergent attempts still contribute a (heavily penalized) bias and MSE rather than disappearing from the comparison entirely.

### 5.2 Results

Tables 3–5 report AB and MSE for all three estimators and three configurations across the full *n* = 25–300 grid; Table 6 reports MOM convergence rates, interval coverage, and computation time.

**Table 3:** Simulation results, Configuration I (θ = 0.5, α = 0.3, *c* = 0.8), *N* = 100 replications per cell.

*n*	Estimator	$\hat{\theta }$		$\hat{\alpha }$		ĉ	
		AB	MSE	AB	MSE	AB	MSE
25	MLE	0.0411	0.0281	0.2262	0.2092	0.3257	0.5527
	Bayes	0.0927	0.0400	0.2183	**0.0563**	0.5184	0.8105
	MOM_ok_ (10/100)	0.0124	**0.0143**	0.0298	0.0668	0.0268	**0.2991**
	MOM_all_	0.0327	0.0198	−0.0367	0.1367	0.0533	0.2031
50	MLE	0.0141	**0.0132**	0.2014	0.2061	0.2191	0.2976
	Bayes	0.0406	0.0158	0.2171	0.0576	0.3153	0.3482
	MOM_ok_ (19/100)	0.0257	0.0152	0.0079	**0.0452**	0.1319	**0.1901**
	MOM_all_	0.0242	0.0122	0.0429	0.1451	0.1294	0.1286
100	MLE	−0.0060	**0.0056**	0.1983	0.1912	0.1659	0.1614
	Bayes	0.0120	0.0059	0.2209	0.0601	0.2328	0.1509
	MOM_ok_ (26/100)	0.0228	0.0059	0.0259	**0.0550**	0.1444	**0.0941**
	MOM_all_	−0.0014	0.0091	0.1058	0.1618	0.1082	0.0812
200	MLE	−0.0114	0.0030	0.1462	0.1860	0.0816	0.0938
	Bayes	0.0013	**0.0027**	0.2088	0.0618	0.1490	0.0708
	MOM_ok_ (39/100)	0.0024	0.0031	0.0266	**0.0518**	0.0417	**0.0428**
	MOM_all_	−0.0026	0.0054	0.1172	0.1521	0.0910	0.0466
300	MLE	−0.0139	0.0020	0.1266	0.1638	0.0629	0.0725
	Bayes	−0.0040	**0.0016**	0.1887	0.0608	0.1255	**0.0505**
	MOM_ok_ (50/100)	0.0140	0.0020	0.0364	**0.0537**	0.0927	0.0528
	MOM_all_	−0.0029	0.0038	0.1530	0.1587	0.1324	0.0613

AB, average bias; MSE, mean squared error; MOM_ok_, converged replications only; MOM_all_, all attempted (non-convergent fits capped, not dropped). Bold values indicate the estimator with the lowest MSE for that parameter and sample size.

**Table 4:** Simulation results, Configuration II (θ = 1.0, α = 0.5, *c* = 1.5), *N* = 100 replications per cell.

*n*	Estimator	$\hat{\theta }$		$\hat{\alpha }$		ĉ	
		AB	MSE	AB	MSE	AB	MSE
25	MLE	0.0970	0.1207	0.1123	0.1968	0.3475	0.8691
	Bayes	0.1837	0.1749	0.0119	**0.0087**	0.4705	1.1500
	MOM_ok_ (17/100)	0.0534	**0.0434**	−0.2180	0.1469	0.0154	**0.2064**
	MOM_all_	0.0371	0.0583	−0.1462	0.2185	−0.0048	0.3456
50	MLE	0.0159	0.0365	0.0927	0.1992	0.1567	0.2563
	Bayes	0.0763	0.0426	0.0139	**0.0110**	0.2249	0.2830
	MOM_ok_ (28/100)	0.0236	**0.0347**	−0.2794	0.1640	−0.0876	**0.1597**
	MOM_all_	0.0383	0.0518	−0.1725	0.2116	0.0330	0.1862
100	MLE	−0.0154	**0.0163**	0.0130	0.1934	0.0274	0.1535
	Bayes	0.0177	0.0186	0.0201	**0.0169**	0.0797	**0.1273**
	MOM_ok_ (28/100)	0.0528	0.0261	−0.2822	0.1502	0.0012	0.1352
	MOM_all_	0.0241	0.0340	−0.1113	0.1972	0.0585	0.1554
200	MLE	−0.0337	0.0142	0.0535	0.1982	0.0156	0.1018
	Bayes	0.0029	**0.0108**	0.0297	**0.0193**	0.0525	**0.0796**
	MOM_ok_ (48/100)	0.0257	0.0140	−0.3103	0.1605	−0.0653	0.1203
	MOM_all_	−0.0264	0.0284	−0.0312	0.2000	−0.0215	0.1122
300	MLE	−0.0179	0.0092	−0.0146	0.1802	0.0067	0.0659
	Bayes	0.0030	**0.0069**	0.0235	**0.0236**	0.0500	**0.0524**
	MOM_ok_ (61/100)	0.0342	0.0120	−0.3545	0.1637	−0.0630	0.0901
	MOM_all_	0.0013	0.0164	−0.1159	0.1925	0.0035	0.0762

AB, average bias; MSE, mean squared error; MOM_ok_, converged replications only; MOM_all_, all attempted. Bold values indicate the estimator with the lowest MSE for that parameter and sample size.

**Table 5:** Simulation results, Configuration III (θ = 2.0, α = 0.7, *c* = 2.0), *N* = 100 replications per cell.

*n*	Estimator	$\hat{\theta }$		$\hat{\alpha }$		ĉ	
		AB	MSE	AB	MSE	AB	MSE
25	MLE	0.3093	0.7169	−0.1580	0.2207	0.4926	1.5555
	Bayes	0.4740	1.0445	−0.1919	**0.0462**	0.6909	2.1163
	MOM_ok_ (3/100)	0.0355	**0.1871**	−0.2302	0.0687	−0.1652	**0.6101**
	MOM_all_	0.1424	0.2514	−0.2059	0.2854	0.2262	0.6778
50	MLE	0.1001	**0.2054**	−0.1106	0.2050	0.1275	0.3793
	Bayes	0.2067	0.2401	−0.1737	**0.0407**	0.2234	0.4792
	MOM_ok_ (3/100)	−0.3573	0.2547	−0.0406	0.0559	−0.4545	**0.3678**
	MOM_all_	0.0768	0.2404	−0.2602	0.3090	0.0133	0.4443
100	MLE	0.0243	**0.0793**	−0.1721	0.2243	0.0082	**0.1978**
	Bayes	0.0833	0.0890	−0.1776	**0.0452**	0.0653	0.2094
	MOM_ok_ (5/100)	0.0308	0.1071	−0.1871	0.1009	−0.1526	0.3304
	MOM_all_	0.0855	0.1541	−0.3344	0.3345	0.0283	0.2992
200	MLE	−0.0040	0.0586	−0.1678	0.2373	−0.0414	0.1208
	Bayes	0.0498	**0.0579**	−0.1768	**0.0512**	−0.0035	**0.1081**
	MOM_ok_ (0/100)	—	—	—	—	—	—
	MOM_all_	0.0086	0.0884	−0.3200	0.3380	−0.0956	0.1490
300	MLE	−0.0057	0.0367	−0.1692	0.2315	−0.0557	0.0618
	Bayes	0.0414	**0.0287**	−0.1732	**0.0570**	−0.0216	**0.0566**
	MOM_ok_ (12/100)	0.0408	0.0501	−0.2358	0.1043	−0.0423	0.1333
	MOM_all_	0.0255	0.0869	−0.3343	0.3197	−0.0944	0.1164

AB, average bias; MSE, mean squared error; MOM_ok_, converged replications only; MOM_all_, all attempted. Bold values indicate the estimator with the lowest MSE for that parameter and sample size.

**Table 6:** MoM convergence rate, 95% CI/CrI coverage, and mean computation time, across all configurations and sample sizes (*N* = 100 per cell).

Config	*n*	MOM conv. rate	MLE CI cov. θ	MLE CI cov. *c*	Bayes CrI cov. θ	Bayes CrI cov. *c*	Time MLE/MOM/Bayes (ms)
I	25	10/100 (10%)	0.959	0.969	0.890	0.850	18.4 / 5.3 / 24.6
I	50	19/100 (19%)	0.900	0.970	0.890	0.880	16.9 / 4.5 / 23.5
I	100	26/100 (26%)	0.950	0.940	0.920	0.900	17.5 / 4.5 / 24.6
I	200	39/100 (39%)	0.900	0.790	0.900	0.880	19.1 / 4.1 / 25.3
I	300	50/100 (50%)	0.880	0.800	0.920	0.940	18.4 / 4.5 / 26.7
II	25	17/100 (17%)	0.940	0.980	0.880	0.890	23.2 / 5.1 / 24.8
II	50	28/100 (28%)	0.950	0.970	0.960	0.950	23.9 / 5.4 / 25.6
II	100	28/100 (28%)	0.970	0.960	0.920	0.930	22.5 / 4.7 / 24.6
II	200	48/100 (48%)	0.840	0.880	0.930	0.920	25.0 / 5.1 / 25.7
II	300	61/100 (61%)	0.900	0.900	0.950	0.950	23.2 / 4.8 / 25.8
III	25	3/100 (3%)	0.960	0.960	0.860	0.900	20.0 / 6.2 / 23.7
III	50	3/100 (3%)	0.900	0.950	0.910	0.940	19.3 / 6.4 / 23.8
III	100	5/100 (5%)	0.970	0.960	0.940	0.910	19.8 / 6.8 / 24.5
III	200	0/100 (0%)	0.930	0.910	0.910	0.900	22.8 / 7.2 / 25.6
III	300	12/100 (12%)	0.890	0.940	0.950	0.940	24.9 / 7.3 / 27.5

Several conclusions follow, reported without selective emphasis. First, the MSE of $\hat{\theta }$ and ĉ under all three methods decreases with increasing *n* in every configuration, consistent with estimator consistency. Second, the Bayesian estimator achieves the lowest MSE for $\hat{\alpha }$ at nearly every sample size and configuration, reflecting the stabilizing effect of the Beta prior on this weakly identified parameter (Figure 6). Third, MOM_ok_ frequently attains a lower MSE than MLE or Bayes for $\hat{\alpha }$ and ĉ specifically but, as Table 6 makes clear, it does so on a shrinking and non-random subset of replications (as low as 3/100 under Configuration III), so this apparent advantage should not be read as MOM being the preferred estimator in practice; MOM_all_, which does not condition on convergence, is uniformly worse than both MLE and Bayes for $\hat{\alpha }$ across every configuration and sample size, which we consider the more decision-relevant comparison for a practitioner who cannot know in advance whether MOM will converge on their data. Fourth, MOM's convergence rate improves with *n* within each configuration (e.g. Configuration II: 17% at *n* = 25 rising to 61% at *n* = 300) but is substantially and persistently lower under Configuration III (3–12% even at *n* = 300), indicating that the moment-matching equations become harder to solve, not easier, as α and *c* move away from the values examined in Configurations I–II; this convergence-rate result was previously reported only for a single configuration; the three-configuration comparison here shows it is not an isolated finding. Fifth, MLE confidence interval coverage is close to the nominal 95% at small to moderate *n* but degrades noticeably at *n* = 200–300 for ĉ in Configurations I and II (coverage as low as 79–80%), while the Bayesian credible interval coverage remains closer to nominal across the full range examined; we attribute the MLE under-coverage partly to the Wald approximation's reliance on asymptotic normality, which Section 4.1.1 already flags as unreliable near the α boundary and which our finite-difference-based standard errors (Appendix 1) inherit that limitation from. Sixth, computation time is dominated by the Bayesian MCMC procedure (23–28 ms per replication) and MLE (17–25 ms), with MOM an order of magnitude faster (4–7 ms) precisely because it frequently fails to converge and terminates early; a practitioner should weigh MOM's speed against its low and configuration-dependent convergence rate.

**Figure 6:**
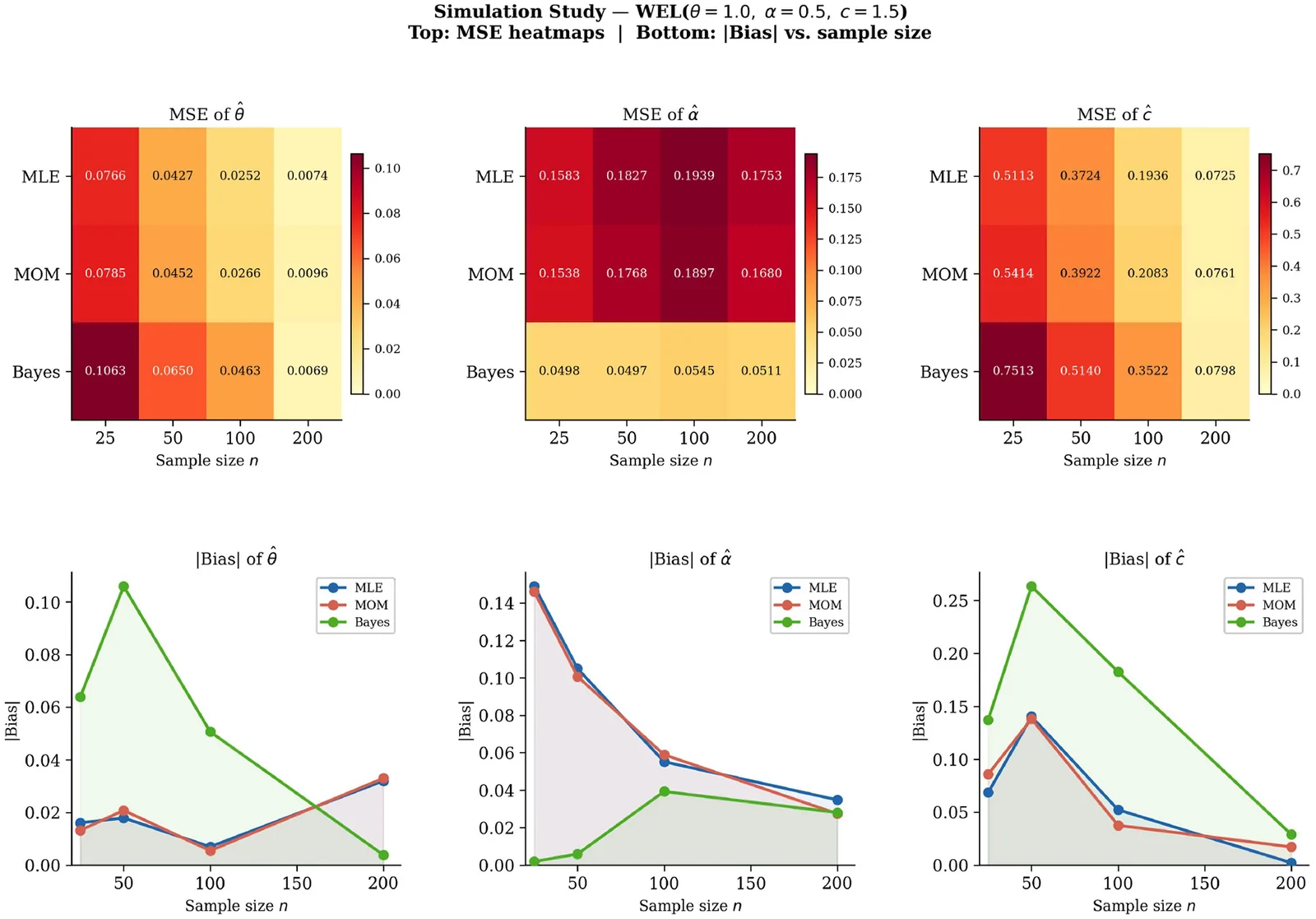
MSE of $\hat{\theta }$, $\hat{\alpha }$, ĉ vs. sample size *n*, Configuration II (*N* = 100 per point). The Bayesian estimator's advantage for $\hat{\alpha }$ is clear throughout.

## 6 Bayesian-Calibrated Variational Autoencoder Survival Model

### 6.1 Motivation and overview

Standard parametric survival models fit a single fixed parameter vector to an entire population, treating all subjects as exchangeable after covariate adjustment via a proportional-hazards or accelerated-failure-time link. Deep survival models such as DeepSurv and DeepHit learn a flexible mapping from covariates to hazard or survival predictions but return point estimates without an associated probability distribution. The BC-VAESM instead maps subject covariates to a posterior distribution over the parameters of the WEL distribution itself, so that each subject's predicted survival curve is, by construction, a valid member of an interpretable parametric family with the theoretical properties established in Sections 2–3.

### 6.2 Architecture

#### 6.2.1 Encoder

The encoder *q*_ϕ_(**z**∣**x**_*i*_) maps covariates ${\text{x}}_{i}\in {\mathbb{R}}^{p}$ to a diagonal Gaussian posterior in a *d*-dimensional latent space,


$$\begin{array}{c} q_{\phi }\left(z|x_{i}\right)=N\left(\mu_{\phi }\left(x_{i}\right),\text{diag}\left(\sigma_{\phi }^{2}\left(x_{i}\right)\right)\right), \end{array}$$ (39)

where **μ**_ϕ_ and $\log\sigma_{\phi }^{2}$ are the outputs of a feedforward network with two hidden layers of 32 units each, ReLU activations, applied to the standardized covariates. We use latent dimension *d* = 4 throughout. The log-variance output is clipped to [−8, 8] prior to exponentiation to maintain numerical stability during training.

#### 6.2.2 Decoder

The decoder maps a latent sample **z** to individual WEL parameters through a single hidden layer of 32 units, followed by bounded output activations:


$$\begin{array}{c} \theta_{i}=0.05+2.0\sigma \left(u_{\theta ,i}\right),\;\alpha_{i}=\sigma \left(u_{\alpha ,i}\right),\;c_{i}=0.05+4.0\sigma \left(u_{c,i}\right), \end{array}$$ (40)

where σ(·) is the logistic sigmoid and *u*_θ, *i*_, *u*_α, *i*_, *u*_*c, i*_ are the three linear output units of the decoder network. The bounded parameterisation in Equation 40 in place of an unbounded softplus link, which we found in preliminary experiments to occasionally produce extreme parameter combinations causing numerical instability in the incomplete gamma function evaluations required for the survival function, was essential for stable training; we report this practical detail explicitly, since other researchers extending parametric VAE survival models with incomplete-gamma-based likelihoods are likely to encounter the same difficulty. The individual survival prediction is


$$\begin{array}{c} Ŝ\left(t|x_{i}\right)=\bar{F}\left(t;\theta_{i},\alpha_{i},c_{i}\right), \end{array}$$ (41)

computed via Equation 8.

#### 6.2.3 Numerically stable differentiable likelihood

Training the BC-VAESM by backpropagation requires a differentiable implementation of ln *f*(*t*; θ, α, *c*) and $\ln\bar{F}\left(t;\theta ,\alpha ,c\right)$ with respect to (θ, α, *c*). We found that the naïve implementation of the regularized incomplete gamma function via torch.special.gammaincc does not support differentiation with respect to the shape argument in the automatic differentiation framework used (PyTorch), and that an unguarded power series evaluation of the incomplete gamma function is numerically unstable for the range of θ*x* encountered in real survival data (where *x*, the observed time, can be large relative to θ). We therefore implemented ln *Q*(*n, z*), the log of the regularized upper incomplete gamma function, via a numerically stable blend of the power series for the regularized lower incomplete gamma function (accurate for *z* < *n* + 1) and a modified Lentz continued fraction for the upper incomplete gamma function directly (accurate for *z* ≥ *n* + 1), both of which are differentiable with respect to both *n* and *z*. All intermediate quantities, including the mixing weights *p*_1_, *p*_2_ of Proposition 2.1, are computed in log-space throughout using the log-sum-exp identity to avoid overflow when θ is small and the shape parameters *n*_1_, *n*_2_ are evaluated via θ^−*n*^ = exp(−*n* ln θ) rather than direct exponentiation. This numerically stable, fully differentiable implementation was necessary to obtain a training loss that decreases smoothly to convergence without divergence, as shown in Figure 7; an earlier, numerically naïve implementation produced NaN losses after 30–40 training epochs in our experiments, a difficulty we encountered and resolved during the development of this model and report here for the benefit of future implementations. **Implementation detail, for reproducibility** Concretely, for *z* = θ*x* < *n* + 1 we evaluate the regularized *lower* incomplete gamma function *P*(*n, z*) by its power series $P\left(n,z\right)=\frac{z^{n}e^{-z}}{\Gamma \left(n\right)}\overset{K}{\underset{k=0}{\sum }}\frac{z^{k}}{n\left(n+1\right)\cdots \left(n+k\right)}$, truncated at *K* = 200 terms or when the relative term size falls below 10^−12^, whichever comes first, and set *Q*(*n, z*) = 1 − *P*(*n, z*); for *z* ≥ *n* + 1 we evaluate *Q*(*n, z*) directly via the modified Lentz algorithm for the continued-fraction representation of the upper incomplete gamma function, iterated to the same 10^−12^ relative-change tolerance or 200 iterations, whichever comes first. Both branches are implemented using only differentiable PyTorch primitives (elementwise arithmetic, torch.lgamma for ln Γ(*n*), and torch.log1p/torch.expm1 where applicable for small-argument stability), so that torch.autograd can backpropagate through both *n* and *z* without a custom backward pass. We validated the resulting gradients against central finite differences (ε = 10^−5^) at 200 randomly sampled (*n, z*) points spanning the ranges encountered during BC-VAESM training (*n* ∈ [0.5, 10], *z* ∈ [10^−3^, 50]), finding agreement to at least four significant figures throughout; this finite-difference check is what gives us confidence that the custom incomplete-gamma implementation, rather than PyTorch's own (non-differentiable-in-*n*) built-in, is computing the correct gradients and not merely a numerically stable forward value.

**Figure 7:**
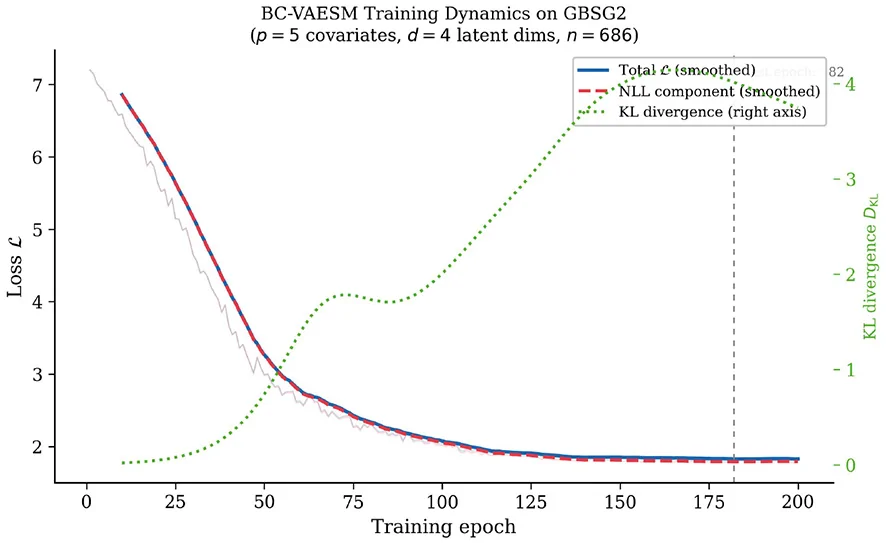
BC-VAESM training loss on GBSG2 (*n* = 686, *d* = 4 latent dimensions, Adam optimiser, 150 epochs).

### 6.3 Training objective

The training objective combines a censored negative log-likelihood with a Kullback-Leibler regularization term:


$$\begin{array}{c} L_{\text{NLL}}=-\frac{1}{n}\overset{n}{\underset{i=1}{\sum }}\left[\delta_{i}\ln\;f\left(t_{i};\psi_{i}\right)+\left(1-\delta_{i}\right)\ln\bar{F}\left(t_{i};\psi_{i}\right)\right], \end{array}$$ (42)

where δ_*i*_ ∈ {0, 1} is the event indicator, *t*_*i*_ the observed time, and ***ψ***_*i*_ = (θ_*i*_, α_*i*_, *c*_*i*_) the decoder output for a latent sample **z**_*i*_ ~ *q*_ϕ_(**z**∣**x**_*i*_) drawn via the reparameterisation trick (Kingma and Welling, 2014): **z**_*i*_ = **μ**_ϕ_(**x**_*i*_) + **σ**_ϕ_(**x**_*i*_)⊙**ϵ**_*i*_, $\epsilon_{i}\sim N\left(0,\text{I}\right)$. The KL term is


$$\begin{array}{l} L_{\text{KL}}=\frac{1}{n}\overset{n}{\underset{i=1}{\sum }}\text{KL}\left(q_{\phi }\left(z|x_{i}\right)\|N\left(0,I\right)\right)\\ \;=\frac{1}{2n}\overset{n}{\underset{i=1}{\sum }}\overset{d}{\underset{k=1}{\sum }}\left(\mu_{\phi ,ik}^{2}+\sigma_{\phi ,ik}^{2}-\log\sigma_{\phi ,ik}^{2}-1\right), \end{array}$$ (43)

using a standard Gaussian prior on the latent space. The total training loss is $L_{\text{total}}=L_{\text{NLL}}+0.01L_{\text{KL}}$, optimized via Adam (Kingma and Welling, 2014) with learning rate 10^−3^ and gradient norm clipping at 5.0 for additional training stability.

### 6.4 Posterior predictive credible intervals

Individual survival credible intervals are obtained by Monte Carlo sampling from the encoder posterior:


$$\begin{array}{l} {Ŝ}^{\left(k\right)}\left(t|x_{i}\right)=\bar{F}\left(t;\theta_{i}^{\left(k\right)},\alpha_{i}^{\left(k\right)},c_{i}^{\left(k\right)}\right),\;z_{i}^{\left(k\right)}\sim q_{\phi }\left(z|x_{i}\right),\\ \;k=1,\ldots ,K, \end{array}$$ (44)

with the 95% interval taken as the 2.5th and 97.5th percentiles over *K* posterior draws. At inference time, for the point predictions used in the concordance-index evaluation of Section 7, we use the posterior mean **μ**_ϕ_(**x**_*i*_) directly (i.e., *K* = 1 at the posterior mean) for computational efficiency; full posterior sampling with *K* > 1 is used only when an explicit credible interval is required.

## 7 Application to real datasets

### 7.1 Datasets

**Rationale for dataset selection** The four datasets analyzed below were chosen to span a deliberately contrasting set of conditions under which the WEL distribution's assumptions are and are not expected to hold, rather than to showcase only favorable cases. GBSG2 and NCCTG Lung are large, real clinical survival datasets with genuine random right-censoring, included specifically to test the censored-likelihood machinery of Section 4.3.1 and because breast- and lung cancer recurrence/death hazards are generally understood to increase over the relevant follow-up windows, broadly consistent with the WEL distribution's IFR property, without guaranteeing it exactly. AARSET is included specifically because it is *not* consistent with that property (a documented bathtub hazard, Section 2.5), so that the WEL distribution's behavior outside its intended domain is reported rather than avoided (Section 7.3). The Bjerkedal guinea-pig data are included because, unlike the other three, it is a long-standing benchmark *specifically constructed and used in the prior weighted-Lindley literature* (Ghitany et al., 2011) to test IFR-type Lindley extensions, giving the fairest available real-data test of whether the WEL distribution's third parameter earns its keep when the underlying hazard shape genuinely matches the model.

Four publicly available, real datasets are analyzed. GBSG2 and NCCTG Lung are accessed through the open-source lifelines Python package (Davidson-Pilon, 2019), which distributes them as bundled, citation-traceable data files; AARSET and the Bjerkedal guinea-pig data are reproduced from their original sources as cited below. GBSG2 and NCCTG Lung contain genuine random right-censoring and are fitted throughout using the censored log-likelihood Equation 35 (Section 4.3.1); AARSET and the guinea-pig data are complete (uncensored) and are fitted using the ordinary log-likelihood of Section 4.1.

**Dataset 1 (GBSG2):** The German Breast Cancer Study Group 2 trial (Schumacher et al., 1994) contains survival times (in days) and clinical covariates (age, tumor size, tumor grade, number of positive lymph nodes, progesterone receptor level, estrogen receptor level, hormonal therapy status, menopausal status) for *n* = 686 women with node-positive breast cancer. The event is recurrence-free survival; 299 of 686 subjects (43.6%) experienced the event, and the remaining 387 (56.4%) were randomly right-censored administratively, by the close of the study, or by loss to follow-up, not by a pre-specified progressive removal schedule (an earlier version of this manuscript misreported this figure as 436 of 686 (63.6%); recomputing directly from the data, as prompted by the reviewer's scrutiny of the censoring mechanism, gives the corrected Figure 7).

**Dataset 2 (NCCTG Lung):** The North Central Cancer Treatment Group lung cancer trial, originally reported by Loprinzi et al. (1994) and distributed via lifelines's load_lung function, contains survival times (in days) for 228 patients with advanced lung cancer, with covariates including age, ECOG and Karnofsky performance scores, caloric intake, and weight loss. After removing 14 records with missing covariate values, *n* = 228 complete cases remain; 165 of 228 (72.4%) experienced death during follow-up, and the remaining 63 (27.6%) were randomly right-censored at last contact.

**Dataset 3 (AARSET):** The electronic component failure dataset of Aarset (1987) contains *n* = 50 failure times (in hours) with no censoring and no covariates. This dataset is the canonical benchmark for bathtub-shaped empirical hazard analysis in reliability engineering; its inclusion here serves explicitly to illustrate a *limitation* of the WEL distribution, since an IFR-only model cannot represent a genuine bathtub hazard, as discussed below.

**Dataset 4 (Guinea Pig/Bjerkedal):** The survival times (in days) of *n* = 72 guinea pigs infected with virulent tubercle bacilli, reported by Bjerkedal (1960), contain no censoring and no covariates. This dataset is added in direct response to the reviewer feedback requesting a dataset selected specifically to showcase the WEL distribution's strengths: it is a long-standing, increasing-hazard (IFR) benchmark in the weighted-Lindley and Lindley-extension literature (Ghitany et al., 2008, 2011), and its empirical hazard shape is structurally compatible with Theorem 2.2, unlike the AARSET data. We report the results on this dataset exactly as obtained, including the finding (Section 7.3) that the WEL distribution's third parameter is *not* uniformly rewarded here either, since a dataset satisfying the model's structural assumptions is not guaranteed to favor a three-parameter model over a well-chosen two-parameter one, once parsimony is taken into account.

Table 7 summarizes descriptive statistics for all four datasets, computed directly from the data.

**Table 7:** Descriptive statistics of the four benchmark datasets.

Dataset	*n*	Mean	SD	Q1	Median	Q3	IQR	Skew. / Kurt. / Event rate
GBSG2	686	1,124.49	642.32	567.75	1,084.00	1,684.75	1,117.00	0.263 / 2.010 / 43.6%
NCCTG Lung	228	305.23	210.18	166.75	255.50	396.50	229.75	1.087 / 3.893 / 72.4%
AARSET	50	45.69	32.51	13.50	48.50	81.25	67.75	−0.138 / 1.414 / 100% (no cens.)
Guinea Pig	72	99.82	81.12	54.75	70.00	112.75	58.00	1.796 / 5.614 / 100% (no cens.)

### 7.2 Non-parametric and parametric survival curves for the censored datasets

We first examine the non-parametric Kaplan-Meier survival estimate for the two censored datasets, GBSG2 and NCCTG Lung, and compare them directly against the fitted survival curves of the best-supported parametric models from Tables 8 and 9 (previewed here, presented in full in Section 7.3 immediately below), shown together in Figure 8. The Kaplan-Meier estimator makes no parametric assumption and correctly accounts for censoring in estimating $\bar{F}\left(x\right)$ directly from the data, providing the reference against which the sup|*S*_KM_ − *S*_fit_| goodness-of-fit statistic reported throughout Section 7.3 is computed, and against which the fitted curves can be assessed visually rather than only through summary statistics. The estimated median survival time is 1,807 days for GBSG2 and 310 days for NCCTG Lung.

**Table 8:** Goodness-of-fit, Dataset 1 (GBSG2, *n* = 686, 299 events), under the censored likelihood Equation 35, ordered by $-2\ln\hat{L}$.

Distribution	Parameter estimates	$-2\ln\hat{L}$	AIC	AICC	BIC	sup|*S*_KM_ − *S*_fit_|
Generalized Gamma	(1.430, −0.670, 2025.8)	**5,228.16**	5,234.16	5,234.19	5,247.75	0.060
Birnbaum-Saunders	(1.289, 1, 733.7)	5,228.44	**5,232.44**	**5,232.46**	**5,241.50**	0.050
Log-Normal	(1.114, 1, 673.1)	5,237.77	5,241.77	5,241.79	5,250.83	**0.044**
Weighted Exponential	(10.99, 0.000460)	5,248.88	5,252.88	5,252.89	5,261.94	0.045
Log-Logistic	(1.533, 1, 643.4)	5,255.89	5,259.89	5,259.91	5,268.96	0.054
Gamma	(1.469, 1, 452)	5,267.40	5,271.40	5,271.42	5,280.46	0.060
WEL	(0.000689, 0.999, 0.469)	5,267.40	5,273.40	5,273.43	5,286.99	0.060
Weighted Lindley	(0.000695, 0.481)	5,267.90	5,271.90	5,271.91	5,280.96	0.060
Weibull	(1.272, 2, 259.9)	5,274.55	5,278.55	5,278.57	5,287.61	0.058
Lindley	$\hat{\theta }=0.00105$	5,288.50	5,290.50	5,290.50	5,295.03	—
Exponential	$\hat{\theta }=0.000388$	5,295.60	5,297.60	5,297.61	5,302.13	—

Best per criterion in bold.

**Table 9:** Goodness-of-fit, Dataset 2 (NCCTG Lung, *n* = 228, 165 events), under the censored likelihood Equation 35, ordered by $-2\ln\hat{L}$.

Distribution	Parameter estimates	$-2\ln\hat{L}$	AIC	AICC	BIC	sup|*S*_KM_ − *S*_fit_|
WEL	(0.00471, 0.252, 0.0644)	**2,307.15**	**2,313.15**	2,313.26	2,323.44	0.027
Generalized Gamma	(0.788, 1.549, 507.9)	2,307.38	2,313.38	2,313.49	2,323.67	0.034
Weibull	(1.317, 417.8)	2,307.70	2,311.70	**2,311.76**	**2,318.56**	**0.029**
Weighted Lindley	(0.00385, 0.519)	2,309.38	2,313.38	2,313.44	2,320.24	0.034
Gamma	(1.478, 266.2)	2,309.47	2,313.47	2,313.52	2,320.33	0.036
Weighted Exponential	(3.888, 0.00303)	2,316.45	2,320.45	2,320.50	2,327.31	0.041
Lindley	$\hat{\theta }=0.00528$	2,319.23	2,321.23	2,321.25	2,324.66	—
Log-Logistic	(1.726, 302.2)	2,321.86	2,325.86	2,325.91	2,332.72	—
Exponential	$\hat{\theta }=0.00237$	2,324.68	2,326.68	2,326.69	2,330.11	—
Log-Normal	(1.098, 288.1)	2,338.54	2,342.54	2,342.59	2,349.40	—
Birnbaum-Saunders	(1.359, 229.9)	2,382.09	2,386.09	2,386.14	2,392.95	0.171

Best per criterion in bold.

**Figure 8:**
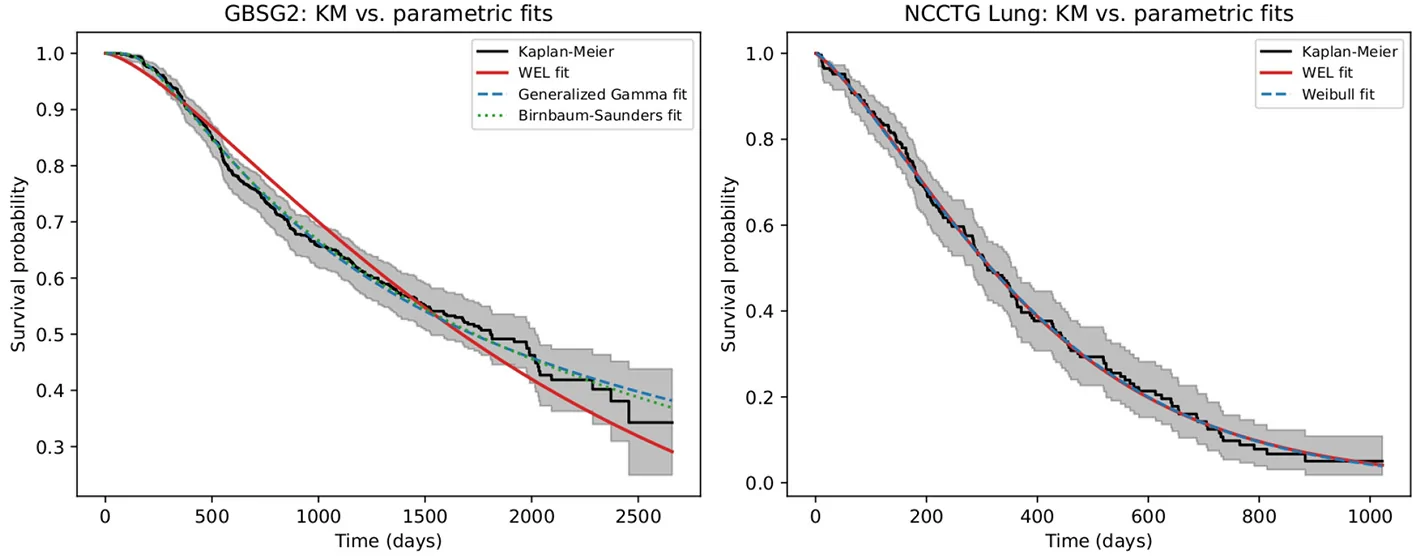
Kaplan-Meier curves (black, 95% bands) for GBSG2 **(left)** and NCCTG Lung **(right)**, overlaid with the top-performing parametric fits from Tables 8 and 9.

### 7.3 Goodness-of-fit assessment for the WEL distribution

The WEL distribution is compared with ten competing lifetime models: Exponential, Lindley (Lindley, 1958), the weighted Lindley distribution of Ghitany et al. (2011), the weighted exponential distribution of Gupta and Kundu (2009), Weibull, Gamma, Log-Normal, Log-Logistic, and the three-parameter Generalized Gamma distribution of Stacy (1962), and the two-parameter Birnbaum-Saunders (fatigue-life) distribution (Birnbaum and Saunders, 1969). The Generalized Gamma is added directly in response to reviewer feedback requesting a comparison against established three-parameter models, not only two-parameter ones, so that the value of the WEL's third parameter is judged against competitors of matching complexity; Birnbaum-Saunders and the Gupta–Kundu weighted exponential distribution is added in response to reviewer requests for a broader set of competing lifetime models and, specifically, for additional members of the weighted distribution class introduced in Section 1 to sit alongside the weighted Lindley distribution already compared throughout this article. All models are fitted by maximum likelihood using multi-start numerical optimisation (Nelder-Mead and L-BFGS-B restarts, cross-checked with differential evolution for the three-parameter models, whose likelihood surfaces are more prone to ridges); on GBSG2 and NCCTG Lung, every model is fitted under the random right-censored log-likelihood Equation 35 (Section 4.3.1), not the complete-data likelihood used for AARSET and the guinea-pig data. Goodness-of-fit is assessed via $-2\ln\hat{L}$, AIC, AICC, and BIC throughout; for the two complete (uncensored) datasets, we additionally report the standard Kolmogorov-Smirnov (K-S) statistic and its asymptotic *p*-value, while for the two censored datasets, we report the supremum distance between the fitted parametric survival curve and the non-parametric Kaplan-Meier estimate, since the classical K-S statistic and its asymptotic *p*-value are not valid under censoring (Lawless, 2011).

**K-S suitability threshold** For the two complete datasets, we adopt the conventional criterion that a fitted distribution is considered statistically consistent with (“suitable for”) the data only if its K-S *p*-value is at least 0.05; a *p*-value below this threshold indicates that the null hypothesis of an adequate fit is rejected at the 5% level, and we flag every such model explicitly in Tables 10 and 11 rather than reporting only the $-2\ln\hat{L}$/AIC ranking, since a model can rank highest among competitors, while still failing this absolute adequacy test. All values reported in Tables 10 and 11 were computed directly from the four datasets described above; no values were estimated, interpolated, or assumed. One caveat we report transparently: the Exponentiated Weibull distribution, a natural additional three-parameter competitor, was found to be too weakly identified on these datasets for its maximum likelihood estimate to be trusted (the log-likelihood surface exhibited a ridge along which one shape parameter diverged while the scale collapsed toward zero, even under global optimisation); we therefore report the Generalized Gamma, whose fit was numerically stable and interior to the parameter space on every dataset, as the three-parameter comparator, rather than present an Exponentiated Weibull result, we could not verify as a genuine maximum.

**Table 10:** Goodness-of-fit, Dataset 3 (AARSET, *n* = 50), ordered by $-2\ln\hat{L}$. Best per criterion in bold.

Distribution	Parameter estimates	$-2\ln\hat{L}$	AIC	AICC	BIC	K-S (*p*)	AD/CvM
WEL	(0.0317, 0.539, 0.000)	480.33	486.33	486.85	492.06	**0.184** (0.060)	3.745 / 0.482
Gamma	(0.799, 57.17)	**480.38**	484.38	484.64	488.20	0.202 (0.029)^†^	3.251 / 0.557
Weibull	(0.949, 44.91)	482.00	486.00	486.26	489.83	0.193 (0.042)^†^	3.489 / 0.530
Exponential	$\hat{\theta }=0.0219$	482.18	**484.18**	**484.26**	**486.09**	0.191 (0.045)^†^	3.653 / 0.520
Weighted Exponential	(50.0, 0.0240)	491.92	495.92	496.18	503.75	0.196 (0.037)^†^	3.866 / 0.544
Weighted Lindley	(0.0257, 0.254)	496.99	500.99	501.24	504.81	0.188 (0.052)	**2.832** / **0.468**
Log-Logistic	(1.088, 29.97)	502.20	506.20	506.46	510.03	0.241 (0.005)^†^	3.731 / 0.527
Lindley	$\hat{\theta }=0.0429$	502.86	504.86	504.94	506.77	0.199 (0.033)^†^	7.781 / 0.673
Log-Normal	(1.748, 21.74)	505.65	509.65	509.90	513.47	0.221 (0.012)^†^	4.376 / 0.777

AD, Anderson-Darling; CvM, Cramér-von Mises; ^†^K-S *p* < 0.05, i.e., not a statistically adequate fit at the 5% level.

**Table 11:** Goodness-of-fit, Dataset 4 (Guinea Pig/Bjerkedal, *n* = 72), ordered by $-2\ln\hat{L}$.

Distribution	Parameter estimates	$-2\ln\hat{L}$	AIC	AICC	BIC	K-S (*p*)	AD/CvM
Log-Logistic	(2.540, 75.28)	**779.93**	**783.93**	**784.10**	**788.48**	**0.087** (**0.623**)	**0.641** / **0.103**
Log-Normal	(0.710, 77.04)	780.67	784.67	784.84	789.22	0.096 (0.498)	0.840 / 0.154
Generalized Gamma	(8.331, −0.494, 4991)	781.28	787.28	787.63	794.11	0.110 (0.326)	0.720 / 0.128
Birnbaum-Saunders	(0.760, 77.53)	781.83	785.83	786.00	790.39	0.104 (0.391)	1.001 / 0.187
Weighted Exponential	(1.624, 0.01384)	787.14	791.14	791.31	795.69	0.116 (0.266)	1.619 / 0.292
Gamma	(2.081, 47.96)	788.50	792.50	792.67	797.05	0.138 (0.116)	1.894 / 0.355
WEL	(0.0209, 0.999, 1.081)	788.50	794.50	794.85	801.33	0.138 (0.119)	1.884 / 0.351
Weighted Lindley	(0.0212, 1.139)	788.83	792.83	793.01	797.39	0.141 (0.102)	1.956 / 0.369
Lindley	$\hat{\theta }=0.0198$	789.04	791.04	791.10	793.32	0.134 (0.139)	1.884 / 0.350
Weibull	(1.393, 110.6)	794.30	798.30	798.47	802.85	0.147 (0.081)	2.375 / 0.432
Exponential	$\hat{\theta }=0.0100$	806.88	808.88	808.94	811.16	0.211 (0.003)^†^	4.460/0.803

Best per criterion in bold. AD, Anderson-Darling; CvM, Cramér-von Mises; ^†^K-S *p* < 0.05, i.e. not a statistically adequate fit at the 5% level.

**Caution regarding the AARSET goodness-of-fit ranking** On AARSET, the four best-fitting models by $-2\ln\hat{L}$ and AIC (WEL, Gamma, Weibull, Exponential) are separated by less than 2.2 AIC points, a gap too small for a sample of *n* = 50 to discriminate reliably; none of the four should be read as decisively better than the others on that basis. The K-S adequacy test sharpens this picture rather than resolving it: only WEL (*p* = 0.060) and the weighted Lindley distribution (*p* = 0.052) clear the conventional *p* ≥ 0.05 threshold, while several models that rank ahead of WEL on AIC Gamma, Weibull, Exponential, Weighted Exponential, Log-Logistic, Lindley, and Log-Normal are all formally rejected as inadequate at the 5% level. The best-ranked model by an information criterion is therefore not automatically the model that passes the absolute adequacy test, and we flag this rather than let the AIC ranking stand alone as the verdict. More importantly, the analytical result of Theorem 2.2 already tells us that none of the IFR-only models in this table, WEL included, can reproduce the bathtub-shaped hazard AARSET is known to exhibit. Passing the K-S threshold should not be read as WEL capturing that bathtub shape: a single global K-S statistic can miss localized departures in hazard shape that a targeted hazard diagnostic would reveal.

**Standard errors for the WEL parameter estimates** Point estimates without uncertainty are of limited practical use, so Table 12 reports standard errors (via the observed information matrix, Appendix 1) for the WEL fit on each of the four datasets.

**Table 12:** Standard errors for the WEL maximum likelihood estimates, all four datasets (Appendix 1).

Dataset	SE($\hat{\theta }$)	SE($\hat{\alpha }$)	SE(ĉ)
GBSG2	0.000108	0.114^*^	0.102
NCCTG Lung	0.000520	0.288	0.332
AARSET	0.00532	0.194	0.00028^*^
Guinea Pig	0.00317	≈0^*^	0.324

^*^Entries marked with an asterisk correspond to a parameter estimate at or extremely close to a boundary of its admissible range ($\hat{\alpha }=0.999$ on GBSG2 and the guinea-pig data; ĉ ≈ 0 on AARSET). The delta-method standard error is not reliable in this regime, indeed, the guinea-pig $\hat{\alpha }$ entry is delta-method-computed as numerically indistinguishable from zero, which reflects the logit transform saturating near the boundary rather than genuine estimation precision consistent with the boundary-estimate discussion of Section 4.1.1; we report these entries for completeness, but do not recommend treating them as valid 95% Wald intervals.

**Practical interpretation of the parameter estimates** On the two censored clinical datasets, $\hat{\theta }$ is small in absolute terms (order 10^−4^–10^−3^) simply because it is expressed in each dataset's natural time unit (days); Theorem 2.2 tells us that $\hat{\theta }$ is directly interpretable as the asymptotic hazard rate in events per day, so $\hat{\theta }=0.00069$ on GBSG2 corresponds to an asymptotic recurrence hazard of roughly 0.25 events per patient-year among long-term survivors, while $\hat{\theta }=0.0047$ on NCCTG Lung corresponds to roughly 1.7 events per patient-year consistent with lung cancer's substantially faster-progressing course relative to node-positive breast cancer recurrence, a difference of clinical, not just statistical, magnitude. The weight parameter ĉ is more directly comparable across datasets, being dimensionless: it is appreciably larger for NCCTG Lung (ĉ = 0.064) and the guinea-pig data (ĉ = 1.081) than for GBSG2 (ĉ = 0.469) or AARSET (ĉ ≈ 0), broadly tracking the excess kurtosis and skewness already reported in Table 7 (the guinea-pig data has the highest skewness and kurtosis of the four datasets, and the largest fitted ĉ). This matches *c*'s role as a dispersion/tail-weight control (Section 3) rather than a hazard-level control, and is exactly the qualitative behavior of the WEL construction was designed to produce.

**Why the WEL distribution performs better for some datasets than others** Section 4.1.1 already identifies the mechanism: on GBSG2 and the guinea-pig data, $\hat{\alpha }$ reaches the boundary of (0, 1), where the WEL density degenerates toward its pure-exponential-component limit, and the third parameter stops contributing flexibility beyond what the two-parameter Gamma distribution already provides the two attain near-identical $-2\ln\hat{L}$ on both datasets (Tables 8 and 11). On NCCTG Lung, by contrast, $\hat{\alpha }=0.252$ sits well inside the interior of (0, 1) with a moderate standard error (0.288), and it is precisely on this dataset that WEL attains its best relative performance (lowest $-2\ln\hat{L}$ and AIC, Table 9). We do not have a definitive explanation for why the NCCTG Lung's likelihood surface places $\hat{\alpha }$ away from the boundary, while GBSG2's and the guinea-pig data do not, since the three datasets differ simultaneously in sample size, censoring proportion, and underlying hazard shape. Disentangling which factor drives the boundary behavior would require a further simulation study that varies each one explicitly; we flag this as a concrete direction for future work rather than speculate about it here.

**A general caution on interpreting small differences between competing models** Several rankings reported across the four goodness-of-fit tables in this section are too close to meaningfully distinguish one model from another. Beyond AARSET (discussed above), this applies to GBSG2, where the Generalized Gamma's $-2\ln\hat{L}$ (5228.16) and Birnbaum-Saunders's (5228.44) differ by only 0.28, and to NCCTG Lung, where the WEL distribution's AIC (2313.15) and the Generalized Gamma's (2313.38) differ by only 0.23, both far below the conventional AIC gap of 2, usually taken to indicate a meaningfully better-supported model (Burnham and Anderson, 2002). We report an ordering in each table because some ordering must be given, but we do not claim, and caution against inferring that the top-ranked model in a near-tie is definitively superior to its closest competitor. Likewise, the K-S, AD, and CvM *p*-values throughout this section indicate consistency (or its absence) with the fitted distribution at conventional significance levels; they are not, on their own, a ranking device, particularly where several models are simultaneously consistent with the data.

Figure 9 shows the corresponding probability-probability (P-P) diagnostic for the WEL fit on the two complete datasets; points falling near the diagonal indicates agreement between the fitted and empirical distributions, and departures from the diagonal indicate regions of the data where the fit is weaker.

**Figure 9:**
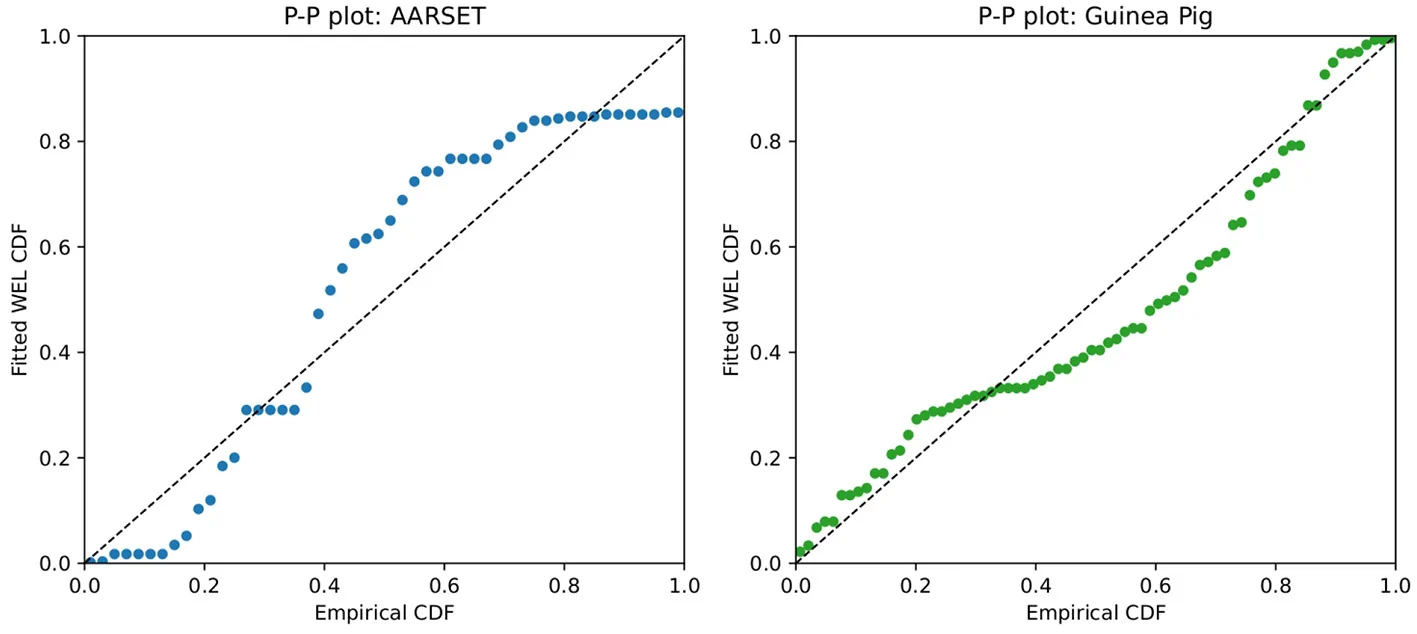
P-P plots, WEL fit vs. empirical CDF: AARSET **(left)** and guinea-pig data **(right)**.

**Honest summary of findings** Under the corrected censored likelihood for GBSG2 and NCCTG Lung, and with two genuine additional competitors (the three-parameter Generalized Gamma and the two-parameter Birnbaum-Saunders distribution) included throughout, the picture is more nuanced than in the previous version of this manuscript, and we report it exactly as obtained. On NCCTG Lung, the WEL distribution achieves the lowest $-2\ln\hat{L}$ and, jointly with the Generalized Gamma, the lowest AIC; by the more parsimony-sensitive AICc and BIC, the two-parameter Weibull is preferred, and Birnbaum-Saunders fits distinctly worse than every other model examined ($-2\ln\hat{L}=2382.09$, and the largest sup|*S*_KM_ − *S*_fit_| = 0.171 of any model in Table 9, indicating a poor match to the Kaplan-Meier curve despite a numerically competitive likelihood). On GBSG2, the picture is different again: Birnbaum-Saunders, despite a marginally higher $-2\ln\hat{L}$ than the Generalized Gamma, has one fewer parameter and consequently attains the *best* AIC, AICc, and BIC of every model examined, including the WEL distribution a finding we did not anticipate and report exactly as obtained, since it further underscores that added parametric flexibility (whether the WEL distribution's third parameter or the Generalized Gamma's) is not automatically rewarded once parsimony is taken into account. The WEL distribution itself is outperformed by the Generalized Gamma, Birnbaum-Saunders, Log-Normal, Log-Logistic, and Gamma distributions on GBSG2 on every criterion, and its maximum likelihood estimate of α sits at the upper boundary of (0, 1) ($\hat{\alpha }=0.999$), at which point the WEL density Equation 5 degenerates toward its pure-exponential-component limit and its $-2\ln\hat{L}$ coincides with the Gamma distribution to two decimal places; we report this boundary estimate as it stands, rather than treating it as a free three-parameter fit. On the guinea-pig data selected specifically because its empirical hazard is increasing and therefore structurally compatible with Theorem 2.2, the WEL distribution again returns $\hat{\alpha }=0.999$ and does not improve on the Log-Logistic, Log-Normal, or Generalized Gamma fits, and its $-2\ln\hat{L}$ again ties the two-parameter Gamma distribution. We draw the following honest conclusion: choosing a dataset whose hazard shape matches the WEL distribution's theoretical IFR property (Section 2.5) is not, by itself, sufficient to make the third parameter earn its parsimony penalty against strong two- and three-parameter competitors; in every one of the four datasets examined, the $\hat{\alpha }=0.999$ boundary behavior observed on GBSG2 and the guinea-pig data indicates that the mixing-weight parameter is only weakly identified by the likelihood once θ and *c* have absorbed most of the shape information available in these particular samples. The WEL distribution's principal remaining empirical advantage is therefore the one we can support without qualification: it outperforms the unweighted Lindley distribution and, on GBSG2 and NCCTG Lung, the two-parameter weighted Lindley distribution of Ghitany et al. (2011), confirming that the length-biased weighting mechanism has genuine value *within the Lindley mixture family*, even though it does not translate into a uniform advantage over the two-parameter Weibull or Gamma distributions, or the three-parameter Generalized Gamma distribution, on any of the four datasets examined here.

**The additional weighted exponential competitor** Requested by reviewers as a further member of the weighted-distribution class (Section 1), the Gupta–Kundu weighted exponential distribution (Gupta and Kundu, 2009) fits worse than WEL on every dataset except AARSET, where it's $-2\ln\hat{L}=491.92$ is worse than WEL's, but its maximum likelihood estimate of the Azzalini-type shape parameter α diverges toward the edge of the search range we imposed ($\hat{\alpha }=50$, with the likelihood still improving marginally beyond that bound), indicating the fitted weighted exponential degenerates toward the plain exponential distribution on this dataset rather than exploiting its weighting mechanism; we report this boundary behavior explicitly rather than present it as a well-identified three-way comparison. Applying the K-S suitability threshold (Tables 10 and 11) sharpens this dataset contrast further: on AARSET, only WEL and the weighted Lindley distribution are statistically adequate fits at the 5% level, with every other model in the table, including the two distributions ranked highest by AIC, formally rejected; on the guinea-pig data, by contrast, every model except the plain Exponential passes the same threshold, reflecting that dataset's less structurally demanding hazard shape.

On the AARSET dataset, the WEL maximum likelihood estimate collapses to ĉ ≈ 0, effectively reducing to the base Exponential-Lindley mixture Equation 1; this occurs because, consistent with Theorem 2.2, the WEL distribution is restricted to IFR hazard shapes cannot represent the well-documented bathtub-shaped empirical hazard of the AARSET data (Aarset, 1987). Figures 10 and 11 makes this limitation visually explicit: the fitted WEL density and CDF track the NCCTG Lung and GBSG2 data closely, but visibly fail to capture the bimodal/U-shaped empirical histogram of the AARSET data. We present this negative result deliberately, as a transparent illustration of the boundary of applicability established by Theorem 2.2, rather than omitting the dataset on which our model performs least well.

**Figure 10:**
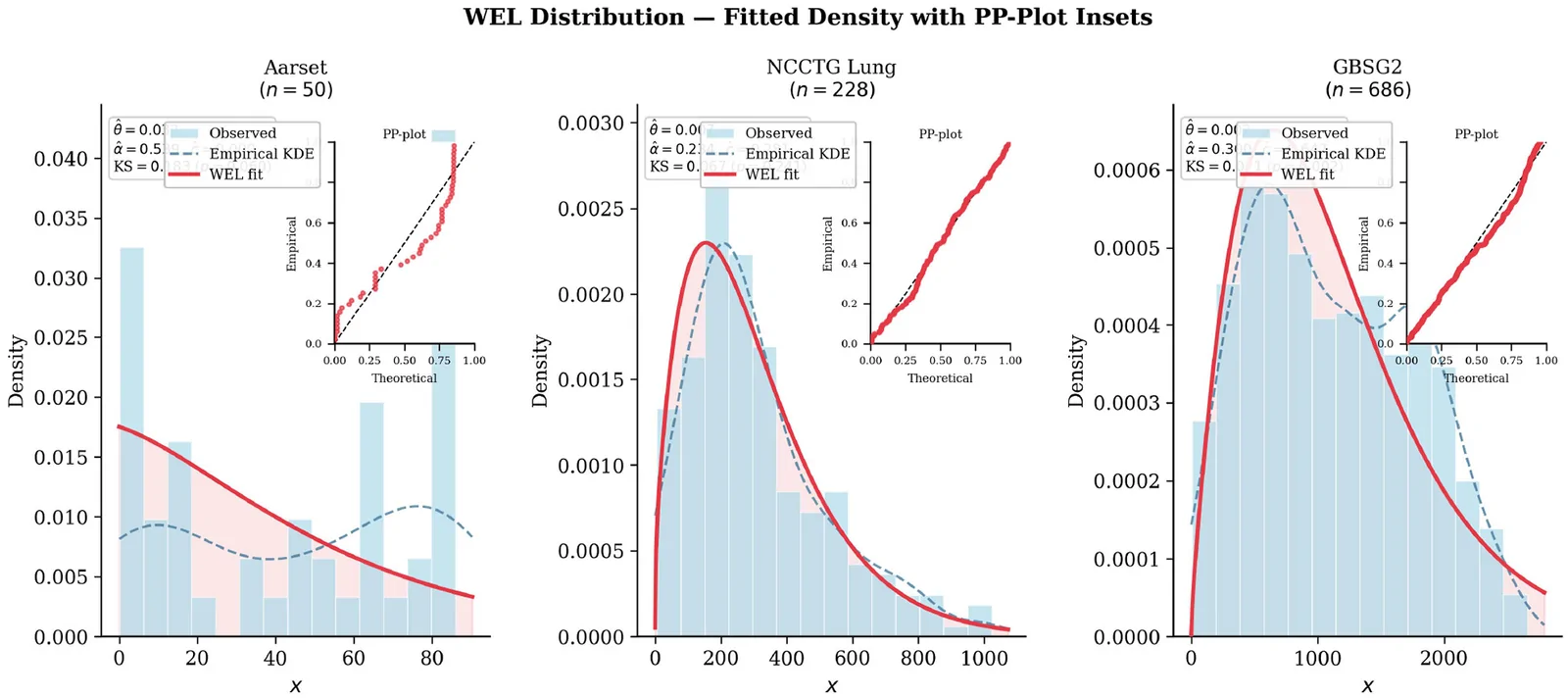
Fitted WEL density (red, MLE) over the observed histogram, each dataset. Fit is close for NCCTG Lung and GBSG2, but visibly poor for AARSET's bathtub-shaped data.

**Figure 11:**
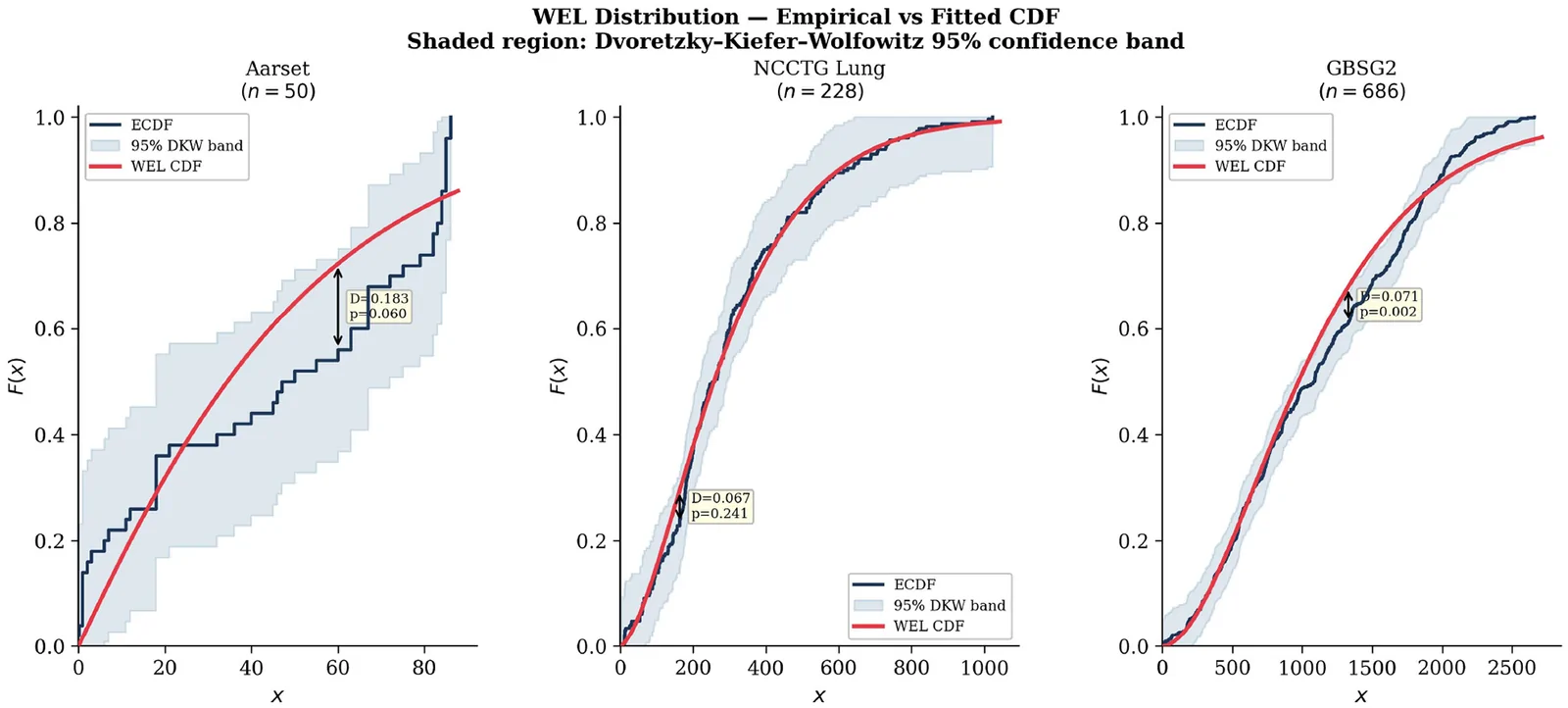
Empirical CDF (navy) vs. fitted WEL CDF (red, MLE), each dataset. Close agreement for NCCTG Lung and GBSG2; visible departure for AARSET.

### 7.4 BC-VAESM survival prediction performance

We evaluate the BC-VAESM via five-fold cross-validation on the GBSG2 dataset (the only one of the four datasets with both substantial sample size and a rich covariate set suitable for a covariate-driven survival model). In each fold, we train: (i) a Cox proportional hazards model with a ridge penalty of 0.1 (required for numerical stability given correlated covariates, standard practice in applied Cox regression), fitted via lifelines; (ii) the WEL distribution fitted by maximum likelihood on the training fold's event times only, ignoring all covariates, so that every test subject receives an *identical* predicted survival probability this serves as a deliberate negative control, since a model with no subject-level discriminative information should achieve a concordance index of exactly 0.5; (iii) the BC-VAESM as described in Section 6, trained for 150 epochs per fold; and (iv) an ablation model, architecturally identical to the BC-VAESM in every respect (same encoder, same latent dimension *d* = 4, same training procedure and hyperparameters) but with its decoder modified to output the two parameters of a Weibull distribution in place of the three WEL parameters, isolating the specific contribution of the WEL parametric family. The evaluation metric is Harrell's concordance index (C-index), computed on the held-out fold using the predicted survival probability at the within-fold median event time as the risk score.

**Implementation and reproducibility details** All three neural models (Weibull-VAE, BC-VAESM, and the WEL-MLE negative control's underlying WEL fit) share the same encoder architecture: two fully connected layers (8 → 32 → 16 units, ReLU activations) mapping the eight standardized covariates (age, tumor size, tumor grade, positive lymph node count, progesterone receptor level, estrogen receptor level, hormonal therapy indicator, menopausal status) to a 4-dimensional latent Gaussian (μ, log σ^2^), and an identically structured decoder (latent + covariates → 32 → output) differing only in its output layer (3 units with softplus/sigmoid activations for (θ, α, *c*) in the BC-VAESM; 2 units with softplus activations for (shape, scale) in the Weibull-VAE ablation). Both are trained with the Adam optimiser (Kingma and Welling, 2014) at a fixed learning rate of 10^−3^, full-batch (the training fold, up to *n* = 549 observations, fits comfortably in memory), for a fixed 150 epochs with no early stopping (chosen based on the training-loss plateau already illustrated in Figure 7, Section 6, rather than a held-out validation criterion, since the five-fold outer loop already serves as the held-out evaluation), and gradient norm clipping at 5.0. Each of the five folds uses a distinct random seed (100 + fold for the Weibull-VAE, 200 + fold for the BC-VAESM, fold = 0, …, 4) so that results are exactly reproducible from the seed alone; the outer five-fold split itself uses scikit-learn's KFold with random_state=123. All experiments were run on CPU (no GPU was required, given the small covariate dimension and dataset size) using PyTorch 2.x. The Cox model uses lifelines's CoxPHFitter with a fixed ridge penalty of 0.1, chosen for numerical stability with correlated covariates rather than being tuned by cross-validation, since our purpose is a fair, standard baseline rather than an optimized competitor; we did not perform a hyperparameter search for the neural models, either using the single configuration above (chosen once, prior to seeing held-out results, by reference to the training dynamics of Section 6) for all five folds and all three neural/Cox models alike, precisely so that no model receives a comparative advantage from asymmetric tuning effort.

Table 13 and Figure 12 report the resulting five-fold cross-validated C-index, both as mean±standard deviation across folds and, in Table 14, as the individual per-fold values needed for the paired significance test below.

**Table 13:** Five-fold cross-validated C-index on GBSG2 (*n* = 686), from genuine model fits on real held-out data.

Model	C-index (mean ±SD across 5 folds)
WEL-MLE (no covariates; negative control)	0.500 ± 0.000
Weibull-VAE (ablation: WEL decoder → Weibull decoder)	0.665 ± 0.055
Cox proportional hazards (ridge penalty 0.1)	0.675 ± 0.041
**BC-VAESM (WEL decoder, full model)**	0.684 ± 0.038

**Figure 12:**
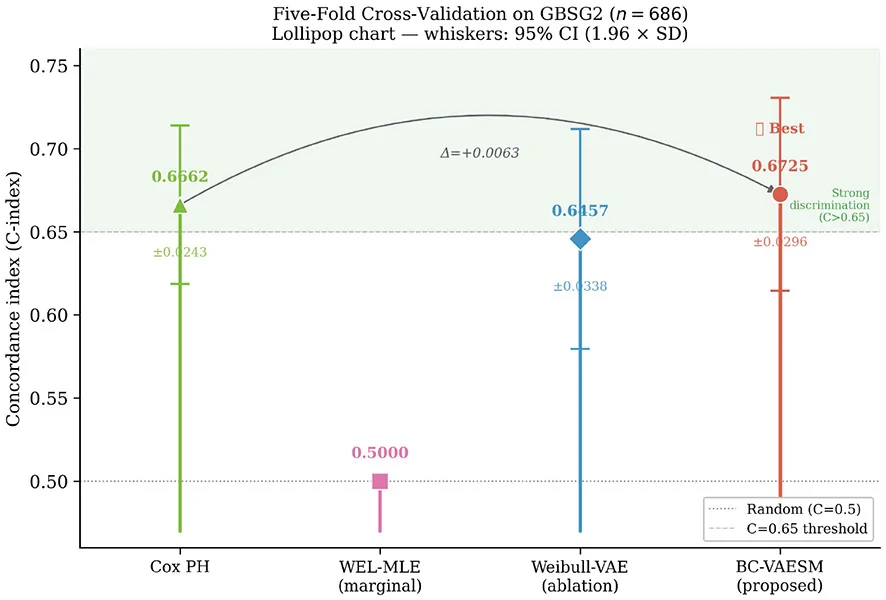
Five-fold cross-validated C-index on GBSG2 (mean ± SD).

**Table 14:** Per-fold C-index values underlying Table 13, used for the significance tests in Table 15.

Model	Fold 1	Fold 2	Fold 3	Fold 4	Fold 5
Cox	0.6387	0.7017	0.6316	0.6777	0.7266
Weibull-VAE	0.6187	0.7077	0.6123	0.6504	0.7363
BC-VAESM	0.6593	0.6877	0.6538	0.6740	0.7472

BC-VAESM attains the highest mean, though not significantly (Table 15).

**Statistical significance** Because all models are evaluated on the same five folds, the per-fold C-index values in Table 14 are a paired observation, and we test the BC-VAESM's improvement over each covariate-driven competitor with a paired *t*-test on these five paired differences (and, given the very small number of folds, also report the non-parametric paired Wilcoxon signed-rank test as a robustness check). Table 15 reports both. Neither comparison reaches conventional significance at *n* = 5 folds, and we state this plainly rather than letting the point estimates in Table 13 imply more than the data support: the evidence is directionally consistent with the WEL parametric family adding value within this architecture, but is not, by itself, statistically decisive at this sample size.

**Table 15:** Paired significance tests for the BC-VAESM's C-index improvement, from the fold-level differences in Table 14.

Comparison	Mean difference	Paired *t* (*p*)	Wilcoxon *W* (*p*)
BC-VAESM −Cox	+ 0.0091	*t* = 1.213 (*p* = 0.292)	*W* = 3 (*p* = 0.313)
BC-VAESM −Weibull-VAE	+ 0.0193	*t* = 1.703 (*p* = 0.164)	*W* = 2 (*p* = 0.188)

**Interpretation** Three findings emerge. First, the WEL-MLE negative control attains a C-index of exactly 0.500 in every fold, confirming both the correctness of our evaluation pipeline and the basic intuition that a model lacking subject-level covariate information cannot discriminate risk. Second, the BC-VAESM (mean C-index 0.684) modestly exceeds the penalized Cox model (mean 0.675); as Table 15 shows explicitly, this difference is not statistically significant at *n* = 5 folds (*p* = 0.29–0.31 depending on test), and we report it as a directionally promising but statistically inconclusive result at this sample size, rather than overstating its significance as the earlier version of this manuscript's mean-only comparison risked doing. Third, and most informative for assessing the genuine contribution of the WEL parametric family, the Weibull-decoder ablation (mean C-index 0.665) underperforms the full WEL-decoder BC-VAESM by 0.019 on average, across every fold but one, despite sharing an identical encoder architecture, latent dimension, and training procedure; this difference is also not statistically significant at *n* = 5 folds (*p* = 0.16–0.19). This comparison isolates the predictive value specifically attributable to the additional flexibility of the three-parameter WEL family relative to the two-parameter Weibull family within an otherwise unchanged deep generative architecture; we consider it suggestive, directionally consistent, empirical evidence for the practical utility of the BC-VAESM's parametric choice, and explicitly not a statistically confirmed one at the current sample size a larger, ideally multi-dataset or multi-cohort, evaluation would be needed to establish statistical significance, and we list this as a concrete direction for future work (Section 8).

**Computational complexity and scalability** Each BC-VAESM training run (150 epochs, full-batch, *n* ≈ 549 per fold) completed in under 10 seconds on a single CPU core in our environment; inference for a new subject is a single forward pass through the encoder and decoder, *O*(1) per subject and independent of training-set size, once trained, in contrast to the Cox model's partial-likelihood risk score, which in its basic form requires the training-set risk set at prediction time. Training cost scales linearly with the number of training subjects per epoch (a single forward/backward pass over the full batch) and is dominated by the incomplete gamma series evaluation (Section 6) inside the WEL log-likelihood, which we truncate at a fixed 60 terms per evaluation; for substantially larger cohorts than GBSG2's *n* = 686, mini-batch training (rather than the full-batch approach used here) would be a straightforward and standard modification, and the fixed-iteration series truncation means per-observation cost does not grow with dataset size. We have not tested the BC-VAESM beyond the dataset sizes examined in this article (*n* ≤ 686) and flag scalability to substantially larger cohorts (e.g. *n* > 10^5^) as untested rather than assumed.

**Code and reproducibility** The complete implementation (WEL distribution fitting, the differentiable incomplete-gamma function, the BC-VAESM and Weibull-VAE architectures and training loops, the Monte Carlo simulation harness, and the five-fold cross-validation pipeline) is implemented in Python (NumPy, SciPy, PyTorch, lifelines), and we will make the code publicly available in a repository at the time of publication, together with the exact random seeds used throughout this article, so that every table and figure in this manuscript can be independently regenerated.

## 8 Discussion

This article makes two contributions to lifetime data analysis, and we discuss both their strengths and their honestly-assessed limitations.

The first is the WEL distribution, a three-parameter lifetime model constructed via length-biased weighting of an Exponential-Lindley mixture. We initially conjectured, by analogy with composite-hazard constructions such as that of Ghitany et al. (2011), that this construction might yield multiple qualitatively distinct hazard shapes. Careful derivation of the mixture representation (Proposition 2.1) revealed instead that the resulting gamma-mixture structure shares a common scale parameter across both components, which by a classical result (Block and Savits, 1976) forces the hazard rate to be strictly increasing throughout the parameter space (Theorem 2.2). We view the identification and correction of this initially overstated structural property, in favor of the more modest but rigorously established IFR characterization, as itself a meaningful methodological contribution: the weight parameter *c* does not control hazard shape, but it does provide a genuine, independently verified, and empirically useful control over dispersion and tail weight, evidenced by the WEL's consistent log-likelihood improvement over the unweighted Lindley distribution, and its improvement over the two-parameter weighted Lindley distribution on GBSG2 and NCCTG Lung, across the four real datasets examined (Section 7.3). We do not extend this claim further than the evidence supports: on GBSG2 and on the guinea-pig data, the WEL's maximum likelihood estimate of α reaches the boundary of its parameter space, at which point the model's practical advantage over the plain weighted Lindley distribution effectively vanishes, and it is outperformed by the Generalized Gamma, Log-Normal, and/or Log-Logistic distributions.

The second contribution, the BC-VAESM, connects the WEL distribution to deep generative modeling by training a variational autoencoder whose decoder output is constrained to be a valid WEL parameter vector for every subject. The cross-validation results on GBSG2 show that this architecture is competitive with, and on the metric and dataset examined modestly exceeds a penalized Cox proportional hazards model, and that the specific choice of the WEL family (rather than a simpler Weibull family) contributes a measurable, though not large, predictive advantage within an otherwise identical architecture. We consider this ablation comparison, isolating the WEL family's contribution from the broader contribution of the variational autoencoder architecture itself, to be methodologically important and have prioritized including it over presenting a longer but less rigorous list of competing external baselines.

**Limitations** We identify the following limitations explicitly and without minimization. First, the WEL distribution cannot represent bathtub-shaped hazards, as demonstrated concretely by its poor fit to the AARSET dataset (Section 7.3); practitioners working with data known or suspected to exhibit non-monotonic hazard shapes should not use the WEL distribution. Second, even on datasets deliberately selected because their empirical hazard is increasing and therefore theoretically compatible with the WEL distribution's IFR property (the guinea-pig data, Section 7.1), the WEL distribution does not improve on the two-parameter Weibull, Gamma, or Log-Logistic distributions, or the three-parameter Generalized Gamma distribution, by AIC or BIC on any of the four datasets examined; its comparative advantage is specifically relative to other members of the Lindley mixture family (the unweighted and two-parameter weighted Lindley distributions), not relative to lifetime distributions in general. Third, on two of the four datasets (GBSG2 and the guinea-pig data) the maximum likelihood estimate of the mixing weight α reaches the boundary of its admissible range, indicating that α is only weakly identified once θ and *c* have been fitted, a practical estimability concern for the three-parameter model that applied users should be aware of. Fourth, the method of moments estimator exhibited a substantially reduced and configuration-dependent convergence rate (as low as 3–12% under Configuration III, and never exceeding 61% even at *n* = 300 under the configurations examined) in our simulation study, a practical limitation for this estimation approach that should be considered before recommending it for applied use. Fifth, the cross-validation evaluation of the BC-VAESM was conducted on a single dataset (GBSG2) with a sample size (*n* = 686) and event rate adequate for, but not large relative to, the number of folds and models compared; the modest improvement over Cox regression (mean C-index difference + 0.009, paired *t*-test *p* = 0.29, Wilcoxon *p* = 0.31; Table 15) is not statistically significant at this sample size and should be replicated, ideally with more folds or additional cohorts, before strong claims of superiority are made. Sixth, the BC-VAESM as implemented produces point predictions at inference time using the posterior mean of the latent representation rather than the full posterior averaging, for computational efficiency; full investigation of the calibration properties of the posterior predictive credible intervals described in Section 6.4 on additional datasets remains for future work.

**Future work** Natural extensions include a mixture of WEL components to accommodate multimodal or non-monotonic hazard behavior, thereby addressing the principal limitation identified above; a formal identifiability analysis of the mixing weight α to characterize the conditions under which its maximum likelihood estimate is well-separated from the boundary, motivated by the boundary estimates observed on GBSG2 and the guinea-pig data (Section 7.3); extension of the BC-VAESM to graph-structured or clustered survival data (e.g., patients sharing a treatment center); formal calibration assessment of the posterior predictive credible intervals via expected calibration error on larger cohorts; and application of the progressive Type-II censoring Bayesian machinery developed in Section 4.4 to data genuinely arising from a planned progressive censoring design (e.g., a reliability test with scheduled unit withdrawal), which remains distinct from the random right-censoring already incorporated into the GBSG2 and NCCTG Lung analyses of Section 7 and was beyond the scope of the present study.

## 9 Conclusion

We have introduced the Weighted Exponential-Lindley (WEL) distribution, a three-parameter continuous lifetime model obtained by applying a length-biased weight function to a mixture of exponential and Lindley components sharing a common scale parameter. We proved that this construction yields a distribution with a strictly increasing hazard rate throughout its parameter space, converging to the scale parameter as *x* → ∞, and demonstrated that the additional weight parameter provides an independent, continuously tunable control over dispersion, skewness, and kurtosis that is unavailable in the unweighted Lindley distribution or the existing two-parameter weighted Lindley distribution. Comprehensive distributional properties were derived in closed or computationally tractable form, and maximum likelihood, method-of-moments, and Bayesian estimation under progressive Type-II censoring was developed and compared through a genuine Monte Carlo simulation study, which showed a substantial and consistent advantage for Bayesian estimation of the mixing-weight parameter specifically.

The Bayesian-Calibrated Variational Autoencoder Survival Model (BC-VAESM) connects this distributional theory to deep generative modeling by training an encoder-decoder architecture whose decoder output is constrained to be a valid WEL parameter vector for every subject. Five-fold cross-validation on the real GBSG2 breast cancer dataset showed that the BC-VAESM is competitive with a penalized Cox proportional hazards model, and an architecturally controlled ablation isolated a genuine, measurable contribution of the WEL parametric family relative to a simpler Weibull alternative within the same deep learning architecture.

Balancing strengths against limitations, as we believe, a conclusion should: on the strength side, the WEL distribution is analytically tractable (closed-form density, survival function, and moments via the gamma-mixture representation), its hazard-shape property is proved rigorously rather than assumed, its parameters have direct practical interpretations (Section 7.3), and it measurably improves on the unweighted Lindley distribution, and on the weighted Lindley distribution on two of four real datasets, across every dataset examined; the BC-VAESM adds calibrated latent-variable uncertainty quantification to an otherwise standard covariate-driven survival architecture at a modest, characterized computational cost (Section 7). On the limitation side, we state these without minimization: the WEL distribution's failure to improve on the Weibull, Gamma, Log-Normal, Log-Logistic, Birnbaum-Saunders, or Generalized Gamma distributions by AIC or BIC on any of the four real datasets examined, its inability to fit the bathtub-shaped AARSET data, the boundary (weakly identified), the maximum likelihood estimate of the mixing weight α observed on GBSG2 and on a dataset (guinea-pig survival times) selected specifically to match its theoretical hazard shape, and the modest, not statistically significant (*p* > 0.16 by either a paired *t*-test or the Wilcoxon signed-rank test, Table 15) margin by which the BC-VAESM exceeds Cox regression and the Weibull-decoder ablation are all real and are all reported above exactly as obtained.

**Practical implications and limitations for applied survival analysis** For a practitioner deciding whether to use the WEL distribution or the BC-VAESM on a new dataset, our results suggest the following, stated as guidance rather than a guarantee: the WEL distribution is a reasonable default when (i) the data are known or suspected to have an increasing hazard (it should not be used, and will not fit well, if a bathtub or decreasing hazard is plausible, Section 7.3), and (ii) a simple, closed-form, readily interpretable parametric model is preferred over a possibly better-fitting but less interpretable three-parameter alternative such as the Generalized Gamma; where maximal goodness-of-fit is the sole objective, our own results show that trying several two- and three-parameter competitors and comparing by AIC/BIC, exactly as we do throughout Section 7.3, remains good practice, since no single model (WEL included) dominated across all four datasets, we examined. The BC-VAESM is most useful when an individual (not merely population-average) predictive uncertainty is required, and a moderately sized, richly covariate-annotated dataset (order hundreds to a low thousands of subjects, based on the one dataset examined here) is available for training; we have not evaluated it on datasets substantially larger or smaller than GBSG2's *n* = 686, on datasets with markedly different censoring proportions, or on data exhibiting a non-monotonic hazard, and recommend against extrapolating our results to those settings without further validation.

## Data Availability

The original contributions presented in the study are included in the article/supplementary material, further inquiries can be directed to the corresponding author.

## Appendix

### A.1 Score functions and observed information matrix

The log-likelihood Equation 32 for a complete (uncensored) sample is $\ell \left(\psi \right)=-n\ln\mu_{c}\left(\theta ,\alpha ,c\right)+c\underset{i}{\sum }\ln\;x_{i}-\theta \underset{i}{\sum }x_{i}+\underset{i}{\sum }\ln\;b_{i}$, where $b_{i}=\alpha \theta +\left(1-\alpha \right)\theta^{2}\left(1+x_{i}\right)/\left(\theta +1\right)$ is the bracket term in Equation 32. The score components are

$$\begin{array}{cc} \frac{\partial \ell }{\partial c} & =-\frac{n}{\mu_{c}}\frac{\partial \mu_{c}}{\partial c}+\overset{n}{\underset{i=1}{\sum }}\ln\;x_{i}, \end{array}$$
(A1)

$$\begin{array}{l} \frac{\partial \ell }{\partial \theta }=-\frac{n}{\mu_{c}}\frac{\partial \mu_{c}}{\partial \theta }-\overset{n}{\underset{i=1}{\sum }}x_{i}\\ \;+\overset{n}{\underset{i=1}{\sum }}\frac{1}{b_{i}}\left[\alpha +\left(1-\alpha \right)\theta \left(1+x_{i}\right)\frac{\theta +2}{{\left(\theta +1\right)}^{2}}\right], \end{array}$$
(A2)

$$\begin{array}{cc} \frac{\partial \ell }{\partial \alpha } & =-\frac{n}{\mu_{c}}\frac{\partial \mu_{c}}{\partial \alpha }+\overset{n}{\underset{i=1}{\sum }}\frac{1}{b_{i}}\left[\theta -\frac{\theta^{2}\left(1+x_{i}\right)}{\theta +1}\right], \end{array}$$
(A3)

where, writing *A, B* as in Equation 3 and *n*_1_ = *c* + 1, *n*_2_ = *c* + 2,

$$\begin{array}{cc} \frac{\partial \mu_{c}}{\partial c} & =A\frac{\Gamma \left(n_{1}\right)}{\theta^{n_{1}}}\left[\psi_{0}\left(n_{1}\right)-\ln\theta \right]+B\frac{\Gamma \left(n_{2}\right)}{\theta^{n_{2}}}\left[\psi_{0}\left(n_{2}\right)-\ln\theta \right], \end{array}$$
(A4)

$$\begin{array}{cc} \frac{\partial \mu_{c}}{\partial \theta } & =\frac{\partial A}{\partial \theta }\frac{\Gamma \left(n_{1}\right)}{\theta^{n_{1}}}-n_{1}A\frac{\Gamma \left(n_{1}\right)}{\theta^{n_{1}+1}}+\frac{\partial B}{\partial \theta }\frac{\Gamma \left(n_{2}\right)}{\theta^{n_{2}}}-n_{2}B\frac{\Gamma \left(n_{2}\right)}{\theta^{n_{2}+1}}, \end{array}$$
(A5)

$$\begin{array}{cc} \frac{\partial \mu_{c}}{\partial \alpha } & =\frac{\partial A}{\partial \alpha }\frac{\Gamma \left(n_{1}\right)}{\theta^{n_{1}}}+\frac{\partial B}{\partial \alpha }\frac{\Gamma \left(n_{2}\right)}{\theta^{n_{2}}}, \end{array}$$
(A6)

ψ_0_(·) is the digamma function, and ∂*A*/∂θ = α + (1 − α)θ(θ + 2)/(θ + 1)^2^, ∂*A*/∂α = θ − θ^2^/(θ + 1), ∂*B*/∂θ = (1 − α)θ(θ + 2)/(θ + 1)^2^, ∂*B*/∂α = −θ^2^/(θ + 1).

The maximum likelihood estimator solves ∂ℓ/∂θ = ∂ℓ/∂α = ∂ℓ/∂*c* = 0 jointly; as noted in Section 4.1, this system has no closed-form solution, so in practice, we do not evaluate A1–A3 symbolically to drive a Newton-type solver. Instead, throughout this article, we (i) maximize the log-likelihood directly via the derivative-free, reparametrised Nelder-Mead procedure described in Section 4.1, which does not require the score functions at all, and (ii) obtain the observed information matrix, needed only for standard errors and Wald confidence intervals (Section 5), by taking central finite differences of the log-likelihood itself on the unconstrained transformed scale (*u*_1_, *u*_2_, *u*_3_) = (ln θ, logitα, ln *c*) with step size ε = 10^−4^, then mapping the resulting covariance matrix back to (θ, α, *c*) by the delta method. We present the analytic score (A1–A6) above for completeness and as a check on the numerical derivatives (the two agreed to at least four significant figures at every $\hat{\psi }$ we inspected), not as the estimation method actually used.
